# Age and duration of obesity modulate the inflammatory response and expression of neuroprotective factors in mammalian female brain

**DOI:** 10.1111/acel.14313

**Published:** 2024-09-04

**Authors:** Binnur Eroglu, Carlos Isales, Ali Eroglu

**Affiliations:** ^1^ Department of Neuroscience and Regenerative Medicine Medical College of Georgia, Augusta University Augusta Georgia USA; ^2^ Department of Medicine Medical College of Georgia, Augusta University Augusta Georgia USA; ^3^ Department of Obstetrics and Gynecology Medical College of Georgia, Augusta University Augusta Georgia USA

**Keywords:** adipose tissue, age, astrogliosis, BDNF, brain, diet‐induced obesity, female, GFAP, high‐fat diet, Iba1, IL1β, IL22, IL6, microgliosis, neuroinflammation, neuroprotection, Nrf2, obesity, Sirt1, SOD, TNFα

## Abstract

Obesity has become a global epidemic and is associated with comorbidities, including diabetes, cardiovascular, and neurodegenerative diseases, among others. While appreciable insight has been gained into the mechanisms of obesity‐associated comorbidities, effects of age, and duration of obesity on the female brain remain obscure. To address this gap, adolescent and mature adult female mice were subjected to a high‐fat diet (HFD) for 13 or 26 weeks, whereas age‐matched controls were fed a standard diet. Subsequently, the expression of inflammatory cytokines, neurotrophic/neuroprotective factors, and markers of microgliosis and astrogliosis were analyzed in the hypothalamus, hippocampus, and cerebral cortex, along with inflammation in visceral adipose tissue. HFD led to a typical obese phenotype in all groups independent of age and duration of HFD. However, the intermediate duration of obesity induced a limited inflammatory response in adolescent females' hypothalamus while the hippocampus, cerebral cortex, and visceral adipose tissue remained unaffected. In contrast, the prolonged duration of obesity resulted in inflammation in all three brain regions and visceral adipose tissue along with upregulation of microgliosis/astrogliosis and suppression of neurotrophic/neuroprotective factors in all brain regions, denoting the duration of obesity as a critical risk factor for neurodegenerative diseases. Importantly, when female mice were older (i.e., mature adult), even the intermediate duration of obesity induced similar adverse effects in all brain regions. Taken together, our findings suggest that (1) both age and duration of obesity have a significant impact on obesity‐associated comorbidities and (2) early interventions to end obesity are critical to preserving brain health.

## Graphical abstract

High fat diet (HFD) induces typical obesity phenotype in female mice independent of their age and duration of HFD. Young females partially resist inflammatory processes induced by moderate duration (13 weeks) of HFD/obesity while females in advanced age develop full‐scale inflammation, indicating age as a risk factor for obesity associated comorbidities. When HFD/obesity persists for a long duration (26 weeks), even young females develop full‐scale inflammation underlining the importance of early corrective interventions to preserve cognitive function and overall health.

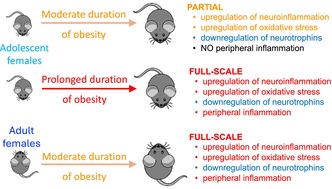

**Abbreviations**
BCA: bicinchoninic acid
BDNF: brain‐derived neurotrophic factor
BMI: body‐mass index
BSA: bovine serum albumin
CLS: crown‐like structure
CNS: central nervous system
DIO: diet‐induced obesity
eCG: equine chorionic gonadotropin
GFAP: glial fibrillary acidic protein
H&E: hematoxylin and eosin
hCG: human chorionic gonadotropin
HFD: high‐fat diet
IACUC: Institutional Animal Care and Use Committee
Iba1: ionized calcium binding adaptor molecule 1
IHC: immunohistochemistry
IKK: inhibitor of κ kinase
IL: interleukin
IL1β: interleukin 1 beta
IU: international unit
JNK: c‐jun N‐terminal kinase
Keap1: Kelch‐like ECH‐Associated Protein 1
LN: liquid nitrogen
NAD: nicotinamide adenine dinucleotide
ND: normal diet
NeuN: neuronal nuclei
NF‐κB: nuclear factor‐kappa B
Nrf2: nuclear factor erythroid 2‐related factor 2
PBS: phosphate‐buffered saline
PBST: PBS containing 0.1% Tween20
PFA: paraformaldehyde
PKR: protein kinase R
p‐Nrf2: phospho‐ nuclear factor erythroid 2‐related factor 2
PVDF: polyvinylidene fluoride
RIPA: radioimmunoprecipitation assay
RNA: ribonucleic acid
ROS: reactive oxygen species
RT‐PCR: real‐time polymerase chain reaction
SDS: sodium dodecyl sulfate
Sirt1: silent information regulator 1; Sirtuin1
SOD: superoxide dismutase
TLR: toll‐like receptors
TNFα: tumor necrosis factor‐alpha
WAT: white adipose tissue
WHO: World Health Organization

## 1 INTRODUCTION

Obesity is a condition characterized by the excessive accumulation and storage of body fat that is known to impair health and reduce the life span (Hotamisligil, 2006). It is recognized as a significant global health problem with increasing prevalence in many countries (Allison et al., 2008). An individual is considered overweight with a body‐mass index (BMI; a ratio of weight to height) between 25.0 and 29.9 kg/m^2^ and obese when a BMI is 30 kg/m^2^ or higher (Seidell & Flegal, 1997). According to the World Health Organization (WHO) estimation in 2016, nearly 2 billion adults worldwide were overweight, and over half a billion were obese (World Health Organization, 2016). In terms of gender, 39% of men and 40% of women aged 18+ were overweight (BMI ≥25 kg/m^2^), and 11% of men and 15% of women were obese (BMI ≥30 kg/m^2^). The prevalence of overweight and obesity among children and adolescents aged 5–19 is also increasing. In 2016, over 340 million children and adolescents were overweight or obese. Regarding gender, 18% of girls and 19% of boys were overweight, and 6% of girls and 8% of boys were obese (World Health Organization, 2016). Reduced physical activity and overconsumption of foods containing highly saturated fats and high carbohydrates appear to be prominent reasons behind this obesity epidemic (Allison et al., 2008; Jeffery & Harnack, 2007; Kanoski & Davidson, 2011).

It has been well recognized that high‐fat diet (HFD) consumption, and thus obesity, leads to chronic inflammation in the central nervous system (CNS), particularly in the hypothalamus, and in other tissues/organs, such as white adipose tissue (WAT), liver, intestine, and muscle (Milanski et al., 2009; Shu et al., 2012; Joshua P. Thaler et al., 2013; Williams, 2012). The activation of toll‐like receptors (TLRs) on immune cells by dietary factors and progressively increased endotoxins (e.g., lipopolysaccharide) in the blood circulation contribute to obesity‐induced inflammation (Kim et al., 2019; Miller & Spencer, 2014). Upon activation, TLRs initiate an inflammatory response by elevating the production of pro‐inflammatory cytokines, such as tumor necrosis factor‐alpha (TNFα), interleukin 1 beta (IL1β), and IL6 (Miller & Spencer, 2014; Shu et al., 2012). During the progression of obesity, hypertrophied adipocytes and immune cells residing in the WAT have also been shown to release pro‐inflammatory cytokines (Elieh Ali Komi et al., 2020). Consequently, diet‐induced obesity (DIO) is associated with low‐grade chronic inflammation, eventually leading to metabolic disorders (Hotamisligil, 2006; Miller & Spencer, 2014; Uysal et al., 1997). After the initial alteration of the energy homeostasis, obesity was also shown to increase the risk of developing chronic diseases, such as diabetes (Boles et al., 2017; Hotamisligil, 2006; Pi‐Sunyer, 2009), cardiovascular diseases (Bastien et al., 2014), reproductive disorders (Rachoń & Teede, 2010), cancer (Fu et al., 2014), and neurodegenerative diseases (Mazon et al., 2017), including psychiatric disorders (Gariepy et al., 2010; Hryhorczuk et al., 2013).

In the CNS, consuming a high‐fat diet seems to increase the expression of pro‐inflammatory cytokines, particularly within the hypothalamus (Dalvi et al., 2017; De Souza et al., 2005; Thaler et al., 2012). The hypothalamus might likely be an early target for inflammation even before substantial weight gain (Dalvi et al., 2017; Thaler et al., 2012). Additionally, high‐fat diets have been linked to the accumulation of microglia and astrocytes in the hypothalamus, triggering inflammatory processes through cytokine production. Microglia can produce ROS when activated, leading to oxidative stress and neuronal dysfunction (Baufeld et al., 2016; De Souza et al., 2005; Sugiyama et al., 2020; Valdearcos et al., 2014; Von Bernhardi et al., 2015). However, further investigation is required to establish the impact of inflammation caused by DIO on other brain regions, such as the cerebral cortex and hippocampus, in a gender‐ and age‐specific manner.

Pro‐inflammatory cytokines, particularly IL1β, may suppress brain‐derived neurotrophic factor (BDNF) in the brain (Guan & Fang, 2006; Lapchak et al., 1993; Tong et al., 2008, 2012). BDNF is crucial in sustaining brain function through its involvement in neurogenesis, synaptic plasticity, and neuroprotection (Lima Giacobbo et al., 2019). Furthermore, BDNF can activate the nuclear factor erythroid 2‐related factor 2 (Nrf2) that helps induce neuronal antioxidant defense (Bruna et al., 2018). Therefore, decreased BDNF levels may compromise prosurvival signaling and potentially cause neuronal death (Lima Giacobbo et al., 2019; Waterhouse & Xu, 2009). Likewise, silent information regulator 1 (aka Sirtuin1, Sirt1), a nicotinamide adenine dinucleotide (NAD)‐dependent histone deacetylase, is known to have neuroprotective effects by activating BDNF, Nrf2, and enzymatic antioxidants such as superoxide dismutases (SODs) (Huang et al., 2015; Jiao & Gong, 2020; Nimmagadda et al., 2013; Xu et al., 2018; Zhao et al., 2019). Proinflammatory cytokines and oxidative stresses associated with DIO may impede the neurotrophic and neuroprotective effects of these molecules in the brain (Chalkiadaki & Guarente, 2012; Guan & Fang, 2006; Lapchak et al., 1993; Morrison et al., 2010; Tong et al., 2008, 2012; Xia et al., 2022) and thus represent potential targets for further research (Boles et al., 2017; Jabri et al., 2017).

HFD studies conducted in rodent models have provided valuable insights into the pathophysiology of human obesity (Collins et al., 2004; Nishikawa et al., 2007). However, most of these studies have only analyzed the impact of HFD on a particular tissue or system at a single time point without considering the subject's age and the duration of obesity. Consequently, a complete understanding of the impact of these variables on obesity is still lacking. Additionally, the current knowledge of DIO is mainly based on studies conducted on male rodents, with limited research on female subjects (Nishikawa et al., 2007) although recent studies indicate significant gender‐specific differences in response to DIO (Chen, Lainez, & Coss, 2021; Hong et al., 2009; Lainez & Coss, 2019; Palmer & Clegg, 2015; Salas‐Venegas et al., 2022; Sanchez et al., 2024). Given the high prevalence of obesity among women (Chooi et al., 2019), it is essential to conduct further research on female subjects to better understand gender‐specific differences in the pathophysiology of obesity.

The objective of this research was to address this gap by studying the effects of an HFD on adolescent (5 weeks old) and mature adult (14 weeks old) female mice for different durations (13 and 26 weeks). The resulting proinflammatory changes (TNFα, IL1β, IL6, IL22, microgliosis, and astrogliosis) and expression of neurotrophic/neuroprotective factors (BDNF, Nrf2, Sirt1, SOD1, and SOD2) were analyzed in different subregions of the brain (hypothalamus, hippocampus, and cerebral cortex) and visceral adipose tissue (inflammation only) and compared to age‐matched controls on a normal diet (ND).

## 2 MATERIALS AND METHODS

### 2.1 Animals and diet

All animal care and use protocols were reviewed and approved by the Institutional Animal Care and Use Committee (IACUC) at Augusta University, and the experiments were carried out in accordance with the IACUC's guidelines (Protocol #2009–0032). All female C57BL/6 wild‐type mice were obtained from The Jackson Laboratory (Bar Harbor, ME) and were housed for a week with a standard rodent diet (Harlan, Teklad, Madison, WI). At 5 weeks of age, 24 female mice were randomly divided into two groups and were fed either a standard normal diet (ND; Teklad 2918, 3.1 kcal/g, 18% kcal from fat, 24% kcal from protein, 58% kcal from carbohydrate), or a high‐fat diet (HFD; Bio‐Serv S3282, 5.49 kcal/g, 59% kcal from fat, 15% kcal from protein, 26% kcal from carbohydrate). In a separate experiment, at 14 weeks of age, 25 mice were randomly divided into two groups and were fed with either ND (*n* = 10) or HFD (*n* = 15), as described above. All mice were group‐housed (3–5 mice/cage) in a temperature‐controlled room (18–23°C) and were kept under a 10‐h dark/14‐h light cycle. Water and their respective diets were available ad libitum, and body weights were recorded weekly. Food intake was measured by subtracting the remaining food from a weighed aliquot every day at 12 weeks of feeding. Energy intake was calculated by multiplying food intake measurements with the kcal per gram of their respective diets (5.49% kcal/g for HFD and 3.1 kcal/g for ND).

### 2.2 Tissue collection

After 13 weeks or 26 weeks of being on the diet, mice were euthanized with CO_2_, and tissues were collected. Since sex steroid hormones can influence expression and signaling of neurotrophins (Bimonte‐Nelson et al., 2004; Cavus & Duman, 2003; Chan & Ye, 2017; Fortress et al., 2014), we attempted to minimize sex steroid‐induced variations by synchronizing the estrous cycle of all female mice through consecutive injections of equine chorionic gonadotropin (eCG) and human chorionic gonadotropin (hCG). Approximately 62 hours before euthanasia, mice were injected intraperitoneally with a combination of 5 IU eCG and 2.5 IU hCG (PG 600, Intervet, Millboro, DE), and 48 h later, they were injected with 7.5 IU hCG alone (Sigma, St Louis, MO). Approximately 14 h after the hCG injection, mice were euthanized using CO_2_ inhalation. Subcutaneous WAT and visceral (peri‐ovarian) WAT were immediately fixed in 4% paraformaldehyde (PFA, Sigma #158127) for histology or snap‐frozen in liquid nitrogen (LN). Brain tissues were either fixed in 4% PFA or dissected into subregions (cerebral cortex, hippocampus, and hypothalamus), snap‐frozen in LN, and stored in a − 80°C freezer.

### 2.3 Histology and immunohistochemistry

Fixed tissues were embedded in paraffin and sectioned in 6‐μm thickness. After paraffin sections were de‐paraffinized in xylene and rehydrated with a series of alcohols, they were either stained with hematoxylin and eosin (H&E) or treated in sodium citrate buffer (10 mM sodium citrate, 0.05% Tween20, pH 6.0) for antigen retrieval. For immunohistochemistry, slides were blocked with 3% bovine serum albumin (BSA) in phosphate‐buffered saline (PBS) for 1 hour at room temperature and incubated overnight at 4°C with the primary antibodies (Iba1 #A1527, BDNF #1307, Nrf2 #A0674, p‐Nrf2 S40 #AP1133, and Sirt1 #A17307, ABClonal; NeuN #MA533103, Invitrogen; SOD2 #66474‐1‐Ig, Proteintech; and GFAP #Z0334, Dako). After washing the slides with PBS containing 0.1% Tween20 (PBST) three times for 10 minutes, they were incubated with the secondary antibody (Alexa Fluor‐555 goat anti‐rabbit IgG, #A‐21428 or Alexa Fluor‐488 goat anti‐mouse IgG, # A11001) for 1 h at room temperature. Slides were washed in PBST, counterstained with Hoechst 33342 (Thermofisher #H1399), and mounted. Both fluorescence and bright field images were captured with a Keyence microscope (BZ‐X710).

To quantify the number of positively immunostained cells per view field, histological sections of the hippocampus, hypothalamus, and cerebral cortex were stained, and images were captured at 20x magnification. The cells were counted using the ImageJ software. The cell count of each group was shown as a fold change relative to that of ND‐13w.

To quantify the size of adipocytes, histological sections of subcutaneous WAT were stained in H&E, and representative images were taken from stained sections at 20x magnification. The area of adipocytes was then measured using the Adiposoft plugin in ImageJ software (Galarraga et al., 2012). To quantify CLS, histological sections of subcutaneous WAT were immunolabeled with Iba1. A CLS was considered present when an adipocyte was surrounded by at least 50% Iba1 labeling.

### 2.4 Western blotting

Brain subregions (hypothalamus, hippocampus, and cerebral cortex) were lysed in ice‐cold radioimmunoprecipitation assay (RIPA) buffer containing 150 mM NaCl, 1% NP‐40, 0.5% sodium deoxycholate, 0.1% SDS, and 50 mM Tris–HCl (pH 8.0) supplemented with 1x protease inhibitors. The concentration of the protein samples was determined with a bicinchoninic acid (BCA) protein assay kit (Pierce). The protein samples were electrophoresed on 8%–10% sodium dodecyl sulfate‐polyacrylamide gel and transferred to a polyvinylidene fluoride (PVDF) membrane (#10600029, GE Healthcare). To reduce nonspecific binding, the PVDF membranes were blocked with 5% (w/v) non‐fat dry milk prepared in PBST for 1 hour at room temperature. The membranes were then incubated with the primary antibodies diluted with 3% BSA in PBST (Nrf2, Sirt1, and β‐Actin; ABClonal; SOD2, Proteintech) at 4°C overnight. After using horseradish peroxidase‐conjugated secondary antibody (#AS014 and #AS003, ABClonal), the immunoreactive bands were visualized using Clarity enhanced chemiluminescent substrate (#1705060, Bio‐Rad) except immunoblotting of SOD2, where a fluorophore‐conjugated goat anti‐mouse secondary antibody (Starbright Blue 700 #12004159, Bio‐Rad) along with a fluorescence scanner (Li‐COR Odyssey) was used. The quantification of the western blot bands was performed using ImageJ software. β‐Actin was used for normalization purposes.

### 2.5 Real‐time polymerase chain reaction (RT‐PCR)

Total RNA was extracted from tissues (brain subregions and peri‐ovarian WAT) and purified using RNeasy mini kit (Qiagen) according to the manufacturer's instructions. cDNA was prepared using a Verso cDNA synthesis kit (ThermoFisher). The signals were detected with SsoAdvanced Universal SYBR Green Supermix (Bio‐Rad) using the LightCycler 96 detection system (Roche) or CFX Opus Real‐time PCR System (Bio‐Rad). The data were analyzed with either the LightCycler 96 SW 1.1 software or CFX Opus Real‐time PCR software and normalized against the housekeeping gene Ppia. The primer sequences are shown in Table S1.

### 2.6 Analysis of superoxide dismutase (SOD)

Both cytoplasmic and mitochondrial fractions were isolated from different brain subregions as described previously (Eroglu et al., 2022). The SOD activity was determined using Superoxide Dismutase Assay Kit (BioAssay Systems) according to the manufacturer's instructions. All samples were assayed in duplicates. The SOD enzyme activity (U/mL) of the samples was calculated from SOD standard curve. SOD activity was then normalized by mg protein amount and expressed as a relative percentage to ND‐13w.

### 2.7 Statistical analysis

All data are presented as the mean ± standard error of the mean (SEM). The data were analyzed using Graph Pad Prism 9.1.0 (GraphPad Software, Inc., San Diego, CA). To compare two groups, two‐tailed independent Student's *t*‐test was used for the most experiments. One‐way ANOVA with Tukey's multiple comparison tests was used to compare differences in adipocyte size and number of CLS between adolescent females fed either ND or HFD for 13 or 26 weeks. The differences between the groups were considered statistically significant when the *p*‐value was <0.05. The level of significance has been indicated in the figure legends as follows: *p < 0.05, **p < 0.01, ***p < 0.001, and ****p < 0.0001.

## 3 RESULTS

### 3.1 Development of HFD‐induced obesity

**FIGURE 1:**
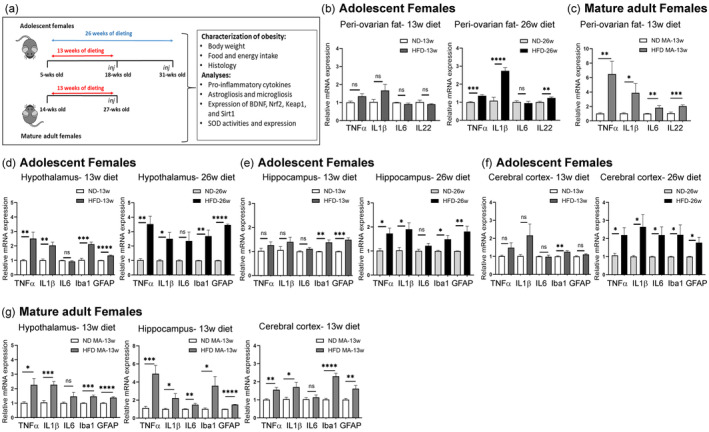
(a) Schematic diagram of the experimental overview. After a week of adaptation period, adolescent (5 weeks old) and mature adult (14 weeks old) females were fed ND or HFD for either 13 or 26 weeks. All mice received intraperitoneal injections (*inj*) of equine chorionic gonadotropin and human chorionic gonadotropin before euthanasia. The obesity phenotype was evaluated by weight gain, food and energy intake, and adipocyte morphology. Additional analyses were also performed, as shown. (b) HFD/obesity‐associated inflammation in visceral adipose tissue in adolescent females after 13 or 26 weeks of dieting, and (c) mature adult females after 13 weeks of dieting. The mRNA levels of proinflammatory cytokines (TNFα, IL1β, IL6, and IL22) were determined in peri‐ovarian fat tissue obtained from ND‐ and HFD‐fed mice. Data are shown as mean ± SEM. ns, non‐significant, *p < 0.05, **p < 0.01, ***p < 0.001, and ****p < 0.0001. (d–f) HFD/obesity‐associated inflammation in different brain subregions of adolescent female mice. Relative mRNA expressions of proinflammatory cytokines (TNFα, IL1β, and IL6), microglia marker (Iba1), and astrocyte marker (GFAP) were analyzed in the hypothalamus (d), in the hippocampus (e), and in the cerebral cortex (f) using RT‐PCR. Data are shown as mean ± s.e.m. ns, non‐significant, *p < 0.05, **p < 0.01, ***p < 0.001, and ****p < 0.0001. (g) HFD/obesity‐associated inflammation in different brain subregions of mature adult female mice. Relative mRNA expressions of pro‐inflammatory cytokines (TNFα, IL1β, and IL6), Iba1, and GFAP were analyzed in the hypothalamus, hippocampus, and cerebral cortex using RT‐PCR. Data are shown as mean ± s.e.m. ns, non‐significant, *p < 0.05, **p < 0.01, ***p < 0.001, and ****p < 0.0001.

To characterize how age and duration of HFD affect the development of an obesity phenotype, adolescent (5 weeks old) and mature adult (14 weeks old) females were fed HFD for either 13 or 26 weeks and evaluated for weight gain, food intake, energy intake, and adipocyte morphology (Figure 1a).

Adolescent female mice subjected to HFD gained significantly more body weight than their control group counterparts. This was observed after 13 weeks (mean ± s.e.m, 37 ± 1.33 g vs. 22.1 ± 0.35 g, *p* < 0.0001) and 26 weeks (43.6 ± 1.03 g vs. 24.6 ± 0.75 g, *p* < 0.0001) of dieting (indicated by black arrows in Figure S1A). The difference in mean body weight was noticeable from the second week on (highlighted by a red arrow in Figure S1A). Notably, the control group on a normal diet also experienced a steady increase in body weight, with a mean growth rate of 0.5 g/week for 13 weeks and 0.34 g/week for 26 weeks of dieting. Nonetheless, these growth rates were much lower when compared to adolescent females on HFD (1.64 g/week and 1.08 g/week, respectively) (Figure S1A,B).

The amount of food consumed by both control and HFD‐fed adolescent female mice was similar (3.25 ± 0.17 vs. 3.19 ± 0.24 g/mouse/day, respectively) (Figure S1C). However, the HFD‐fed animals had significantly higher energy intake at 17.51 ± 1.31 kcal/mouse/day compared to the age‐matched controls at 10.08 ± 0.51 (*p* < 0.001) (Figure S1D).

The size of adipocytes, known to increase with obesity (Elieh Ali Komi et al., 2020), was analyzed through H&E staining. The results showed that adolescent female mice fed an HFD had significantly larger adipocytes in their subcutaneous WAT than those fed a normal diet. These differences were observed after both 13 and 26 weeks of dieting regimens (mean ± s.e.m, 1196 ± 23.6 μm^2^ vs. 4328 ± 333 μm^2^, *p* < 0.001, and 1348 ± 76 μm^2^ vs. 5083 ± 567 μm^2^, *p* < 0.0001, respectively) (Figure S1E,F). However, the prolonged HFD and ND regimens of 26 weeks did not significantly increase the adipocyte size compared to their 13‐week groups (Figure S1F).

The subcutaneous WAT samples taken from adolescent females were also analyzed for the presence of crown‐like structures (CLS), which indicate the accumulation of macrophages around dead or dying adipocytes and are considered one of the characteristic features of inflammation in the adipose tissue (Elieh Ali Komi et al., 2020). A statistically significant increase in CLS was observed when adolescent mice fed HFD for 26 weeks (Figure S1E inserts and S1G).

When mature adult female mice were subjected to HFD for 13 weeks (Figure S2A,B), their body weight was significantly increased compared to control mice fed ND (mean ± s.e.m, 27.8 ± 1.41 g vs. 41.8 ± 1.13 g, *p* < 0.0001) (indicated as a black arrow in Figure S2A). The difference in body weight between the two groups was significant from the start to 13 weeks (*p* < 0.01 for ND‐fed mice and *p* < 0.0001 for HFD‐fed mice). The average growth rate for mice on the ND was 0.41 g/week, while that for mice on the HFD was 1.49 g/week. This was also consistent with a significantly higher energy intake in the HFD group compared to the ND group (mean ± s.e.m, 14.93 ± 0.27 vs. 10.3 ± 0.63 kcal/mouse/day, *p* < 0.001) despite a lower food intake (mean ± s.e.m, 2.7 ± 0.05 vs. 3.1 ± 0.12 g/mouse/day, *p* < 0.05) (Figure S2C,D).

Furthermore, the analysis of the subcutaneous WAT staining showed that mature adult females fed an HFD had significantly larger adipocytes than those of ND‐fed controls (mean ± s.e.m, 4309 ± 172.8 μm^2^ vs. 1553 ± 27.5 μm^2^, *p* < 0.0001) (Figure S2E,F) and significantly increased number of CLS (Figure S2E inset and S2G).

Taken together, our data confirm that the HFD regimens used in the present study induce a typical obesity phenotype in C57BL/6 female mice.

### 3.2 Development of obesity‐associated inflammation in visceral adipose tissue

While obesity is associated with increased production of pro‐inflammatory cytokines like TNFα and IL1β (Elieh Ali Komi et al., 2020), it is less clear how age and duration of HFD affect inflammation in the visceral adipose tissue of female mice. To address this and the temporal relationship between inflammatory processes in visceral fat and neuroinflammation, we examined mRNA expression levels of various inflammatory markers (i.e. TNFα, IL1β, IL6, and IL22) in peri‐ovarian fat pads (Figure 1b). An overview of the experimental outcomes is provided in Table S2.

When subjected to HFD for 13 weeks, adolescent female mice displayed no significant increase in inflammation markers in their peri‐ovarian adipose tissue as compared to their age‐matched control group (Figure 1b). In contrast, adolescent female mice kept on HFD for a prolonged duration of 26 weeks showed a significant increase in the expression of TNFα, IL1β, and IL22, while IL6 remained unchanged (Figure 1b). These findings indicate that inflammation in the visceral adipose tissue of adolescent female mice develops slowly as the duration of HFD extends.

Unlike adolescent females, mature adult female mice fed HFD for 13 weeks showed a significant increase in the expression of TNFα, IL1β, IL6, and IL22 compared to ND‐fed mice (Figure 1c), suggesting that the age significantly influenced the development of inflammation in visceral adipose tissue of female mice.

### 3.3 Development of obesity‐associated inflammation in CNS

To determine how obesity affects neuroinflammation in female mice, we examined the mRNA expression levels of proinflammatory cytokines (TNFα, IL1β, and IL6) and markers of microgliosis (Iba1) and astrogliosis (GFAP) in three distinct brain subregions (hypothalamus, hippocampus, and cerebral cortex) (Figure 1d–f). An overview of the experimental outcomes is provided in Table S2.

In the hypothalamus, a significant increase in the expression level of TNFα, IL1β, Iba1, and GFAP was found upon feeding adolescent females HFD for 13 weeks while the IL6 expression did not significantly change (Figure 1d). After the prolonged duration of HFD for 26 weeks, the hypothalamus of adolescent females displayed a significant increase in the expression level of all inflammatory markers but the increase in the expression level of IL6 still remained insignificant (*p* = 0.056). Taken together, these results suggest that HFD induces inflammatory processes in the hypothalamus earlier than visceral fat tissue.

In the hippocampus of adolescent females (Figure 1e), only expression of Iba1 and GFAP was significantly increased after 13 weeks of HFD, while there was no significant increase in the expression level of TNFα, IL1β, and IL6. In contrast, the prolonged duration of HFD for 26 weeks increased the expression of all pro‐inflammatory markers except IL6 in the hippocampus of adolescent females, underlining the pivotal role of the HFD duration.

The cerebral cortex of adolescent females (Figure 1f) was rather resistant to HFD‐induced proinflammatory processes after 13 weeks and expressed proinflammatory markers similar to ND‐fed controls, except for a significant increase in Iba1 expression. Notably, the prolonged duration of HFD for 26 weeks significantly increased the expression of all proinflammatory markers in the cerebral cortex of adolescent females, indicating again the critical role of persistent obesity.

Unlike adolescent female mice, mature adult females responded to HFD for 13 weeks with significantly increased proinflammatory markers in all three brain regions, although the increase in IL6 expression did not reach a statistically significant level in the hypothalamus and cerebral cortex (Figure 1g). Overall, these results suggest that advanced age may affect the obesity outcome similar to a prolonged duration of obesity in adolescent females.

To confirm HFD‐induced microgliosis, we also performed immunofluorescence staining for Iba1 in various brain sections, including the ventromedial nucleus of the hypothalamus, dentate gyrus of the hippocampus, and M1‐M2 regions of the cerebral cortex. Subjecting adolescent females to HFD for either 13 or 26 weeks led to a significant increase in the number of microglia in the hypothalamus, hippocampus, and cerebral cortex regions with respect to ND‐fed controls (Figure S3A). Notably, the number of microglia further increased after the prolonged HFD for 26 weeks compared to the intermediate‐term HFD of 13 weeks (Figure S3A,B). When assessed based on their morphological phenotypes (Iris et al., 2014; Kreutzberg, 1996), the number of activated microglia was also significantly increased after 26 weeks of HFD (Figure S3C), further indicating inflammatory microgliosis. Upon exposure to HFD for 13 weeks, mature adult females also showed an increase in Iba1‐positive cells in various brain regions, including the hypothalamus, hippocampus, and cerebral cortex (Figure S3D,E).

In addition to mRNA expression, HFD‐induced astrogliosis was also assessed by immunochemistry using GFAP as a marker in hypothalamus, hippocampus, and cerebral cortex. Consistent with the GFAP expression results (Figure 1d–f), the number of astrocytes was significantly increased in the hypothalamus and hippocampus of adolescent females after 13 weeks of HFD while the increase was insignificant in the cerebral cortex (Figure S4A,B). The prolonged HFD of 26 weeks significantly increased the number of astrocytes in all three brain subregions of adolescent females (Figure S4A,B). Similarly, mature adult females displayed significantly increased astrocytes in all three brain subregions after 13 weeks of HFD (Figure S4C,D).

Together, these findings suggest that HFD leads to progressive inflammation in the CNS of female mice, whereas the proinflammatory processes worsen with prolonged HFD and increased age.

### 3.4 Impact of obesity on neurotrophic/neuroprotective factors

#### 3.4.1 Changes in the BDNF level

**FIGURE 2:**
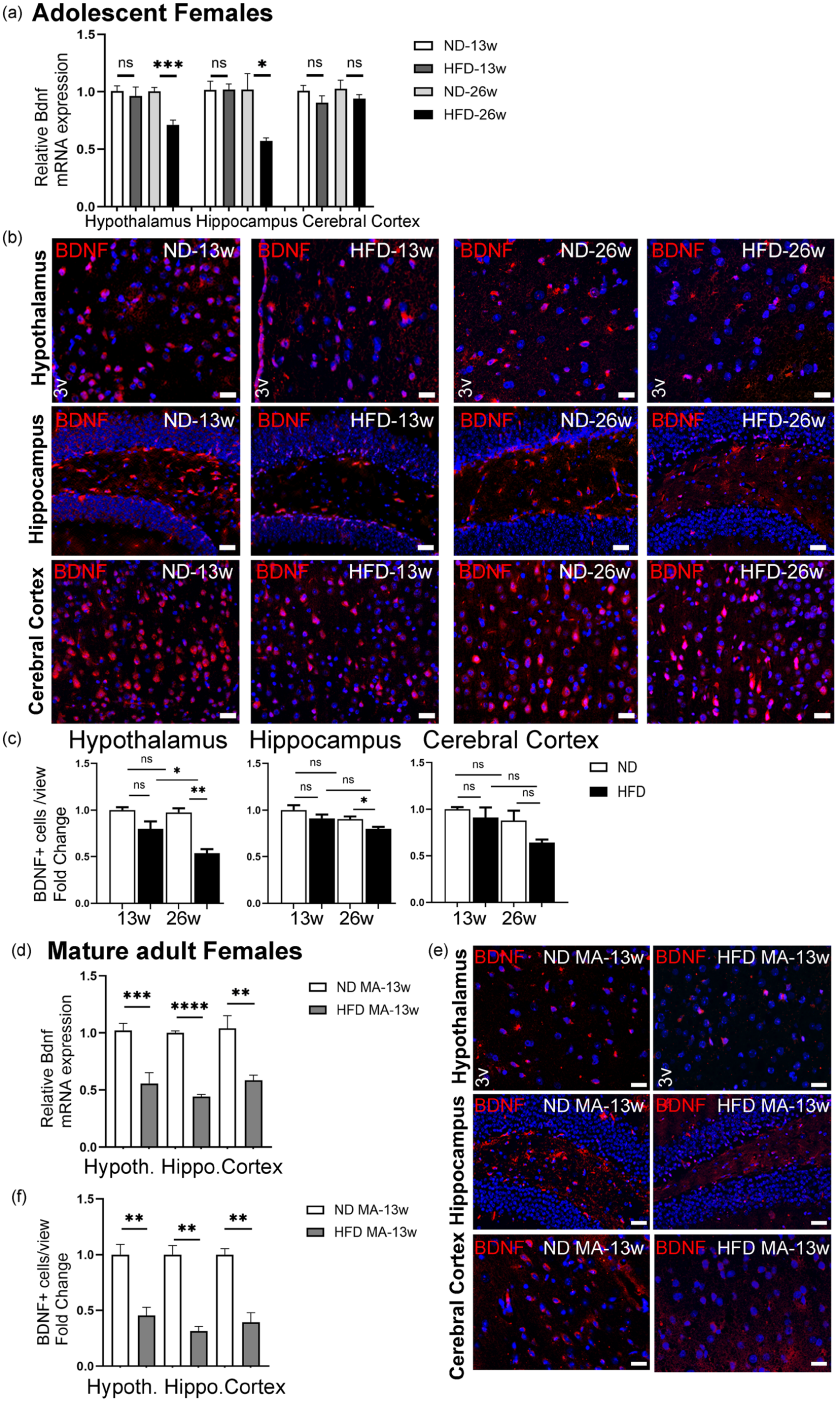
Effect of HFD/obesity on the BDNF level in different brain subregions of female mice. (a) Relative mRNA levels of BDNF in the hypothalamus, hippocampus, and cerebral cortex of adolescent female mice fed either ND or HFD for 13 or 26 weeks. (b) IHC analysis of BDNF expression in the CNS. Representative images of BDNF‐positive cells (in red) and Hoechst‐stained nuclei (in blue) were obtained from the hypothalamus region (3v, third ventricle), the hippocampus region, and the cerebral cortex region from adolescent mice fed either ND or HFD for 13 or 26 weeks. Scale bars: 20 μm. (c) Fold change in BDNF‐positive cell numbers in the hypothalamus, hippocampus, and cerebral cortex regions. Data are shown as mean ± s.e.m. ns, non‐significant, *p < 0.05, and **p < 0.01. (d) Effect of HFD/obesity on the BDNF level in different brain subregions of mature adult (MA) female mice. Relative mRNA levels of BDNF in the hypothalamus, hippocampus, and cerebral cortex following 13 weeks of dieting. (e) IHC analysis of BDNF expression in the CNS. Representative images of BDNF‐positive cells (in red) and Hoechst‐stained nuclei (in blue) were obtained from the hypothalamus region (3v, third ventricle), the hippocampus region, and the cerebral cortex region of mature adult female mice fed either ND or HFD. Scale bars: 20 μm. (f) Fold change in BDNF‐positive cell numbers in the hypothalamus, hippocampus, and cerebral cortex regions of mature adult female mice. Data are shown as mean ± s.e.m. **p < 0.01, ***p < 0.001, and ****p < 0.0001.

To study the influence of HFD on BDNF, we first analyzed mRNA expression levels in the hypothalamus, hippocampus, and cerebral cortex of adolescent female mice. After 13 weeks of HFD, there was no significant change in BDNF expression (Figure 2a). However, the prolonged HFD for 26 weeks led to a significant decrease in BDNF expression in the hypothalamus and hippocampus but not in the cerebral cortex of adolescent females (Figure 2a). To verify these results, we next conducted immunostaining of BDNF in brain sections of adolescent female mice, including the hypothalamus, hippocampus, and cerebral cortex after 13 and 26 weeks of HFD and ND (Figure 2b). Consistent with the mRNA expression findings, the immunostaining results showed no significant change in the number of BDNF+ cells in the hypothalamus, hippocampus, or cerebral cortex after 13 weeks of HFD (Figure 2c). However, after 26 weeks of HFD, the number of BDNF+ cells in the hypothalamus and hippocampus were significantly lower than that of their respective controls while no significant changes were observed in the cerebral cortex region after HFD for 13 or 26 weeks. Overall, these findings indicate that prolonged HFD consumption for 26 weeks reduces BDNF expression and the number of BDNF+ cells, particularly in the hypothalamus and hippocampus of adolescent female mice.

When mature adult females were subjected to HFD for 13 weeks, the expression level of BDNF was significantly lower in all three brain subregions (hypothalamus, hippocampus, and cerebral cortex) compared to those of ND‐fed controls (Figure 2d). These findings were also confirmed by immunostaining results showing that the number of BDNF+ cells were significantly lower in all three brain subregions of HFD‐fed mature adult females when compared to that of their ND‐fed counterparts (Figure 2e,f).

Our co‐labeling experiments with BDNF and neuronal nuclear protein (NeuN), a neuron‐specific marker (Gusel'nikova & Korzhevskiy, 2015), revealed that BDNF is expressed in NeuN+ cells (Figure S5A).

#### 3.4.2 Changes in the Sirt1 level

**FIGURE 3:**
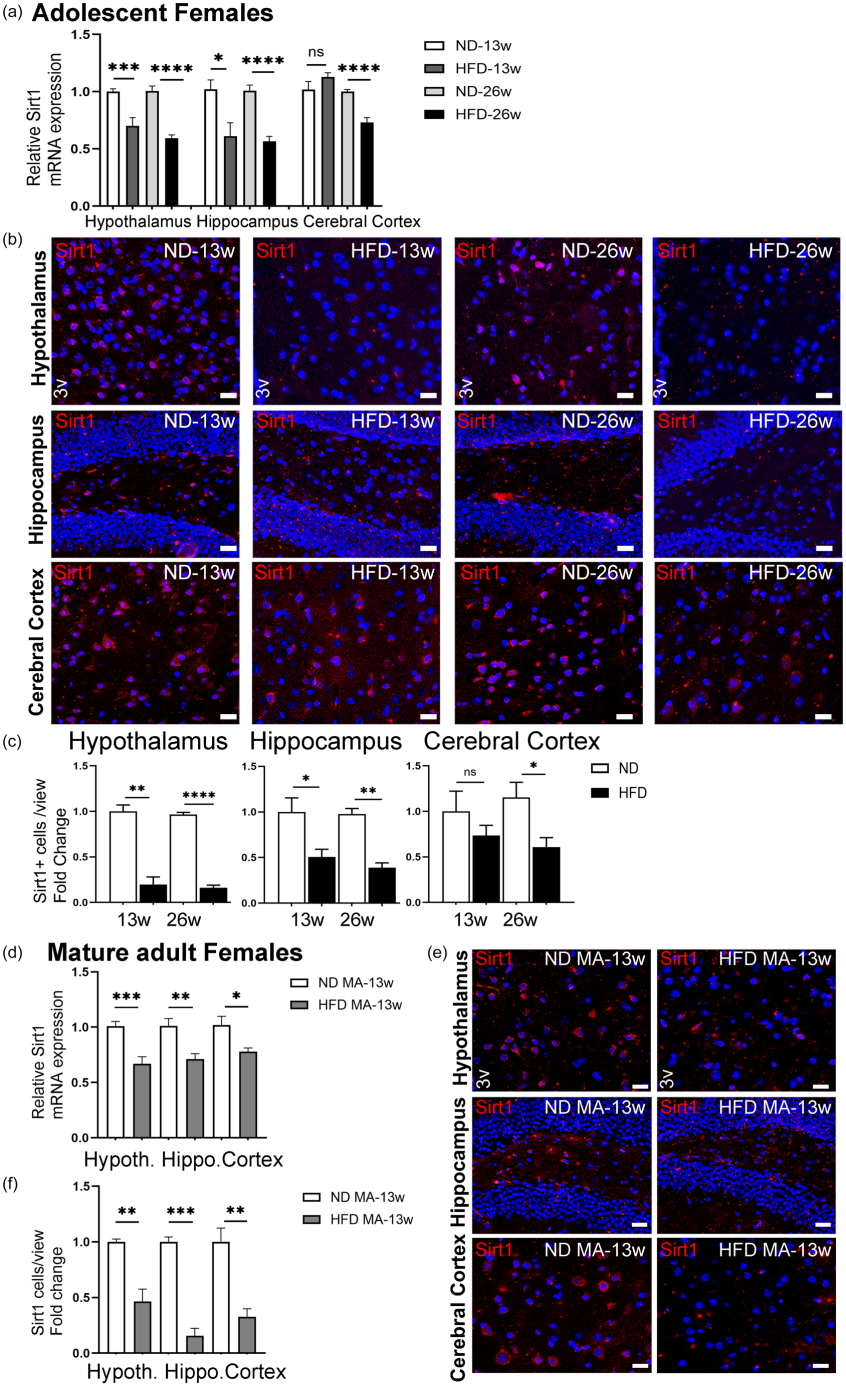
Effect of HFD/obesity on the Sirt1 level in different brain subregions of female mice. (a) Relative mRNA levels of Sirt1 in the hypothalamus, hippocampus, and cerebral cortex of adolescent female mice fed either ND or HFD for 13 or 26 weeks. (b) IHC analysis of Sirt1‐positive cells in the CNS. Representative images of Sirt1‐positive cells (in red) and Hoechst‐stained nuclei (in blue) were obtained from the hypothalamus region (3v, third ventricle), the hippocampus region, and the cerebral cortex region from mice fed an ND or HFD for 13‐weeks or 26‐weeks. Scale bars: 20 μm. (c) Fold change in Sirt1 positive cell numbers in the hypothalamus, hippocampus, and cerebral cortex regions. Data are shown as mean ± s.e.m. ns, non‐significant, *p < 0.05, **p < 0.01, ***p < 0.001, and ****p < 0.0001. (d**)** Effect of HFD/obesity on the Sirt1 level in different brain subregions of mature adult (MA) female mice. Relative mRNA levels of Sirt1 in the hypothalamus, hippocampus, and cerebral cortex following 13 weeks of dieting. (e) IHC analysis of Sirt1 positive cells in different brain subregions. Representative images of Sirt1‐positive cells (in red) and Hoechst‐stained nuclei (in blue) were obtained from the hypothalamus region (3v, third ventricle), the hippocampus region, and the cerebral cortex region from mice fed either ND or HFD for 13 weeks. Scale bars: 20 μm. (f) Fold change in Sirt1‐positive cell numbers in the hypothalamus, hippocampus, and cerebral cortex regions of mature adult female mice. Data are shown as mean ± s.e.m. *p < 0.05, **p < 0.01, and ***p < 0.001.

Sirt1 also acts as a neurotrophic/neuroprotective factor together with BDNF and Nrf2 (Bruna et al., 2018; Caruso et al., 2021; Ding et al., 2016; Huang et al., 2013; Nimmagadda et al., 2013). To assess the effect of HFD on Sirt1, we first analyzed its expression in the hypothalamus, hippocampus, and cerebral cortex subregions of adolescent females after subjecting them to HFD for 13 and 26 weeks. Our results showed that after 13 weeks of HFD, Sirt 1 expression was significantly reduced in the hypothalamus and hippocampus but remained unchanged in the cerebral cortex (Figure 3a). Yet, the prolonged duration (26 weeks) of HFD significantly reduced Sirt1 expression in all three brain subregions, indicating consequential role of obesity duration. These findings were confirmed by the similar outcome of immunostaining results (Figure 3b,c). In terms of Sirt1 protein levels, western blot analyses also yielded statistically similar results after 26 weeks of HFD, although no significant changes were observed after 13 weeks of HFD (Figure S6A,B).

Mature adult female mice subjected to HFD for 13 weeks displayed significantly reduced Sirt1 expression in their hypothalamus, hippocampus, and cerebral cortex when compared to their respective controls (Figure 3d). Our immunochemistry experiments further confirmed these findings by showing fewer Sirt1+ cells in all three brain subregions after 13 weeks of HFD (Figure 3e,f). Our western blot analyses showed that the protein levels of Sirt1 were significantly reduced only in the hypothalamus and hippocampus (Figure S6D,E). Although the Sirt1 protein level in the cerebral cortex was lower in HFD‐fed mice, the difference was not statistically significant.

Sirt1 is also expressed in NeuN+ cells as shown by co‐labeling (Figure S5B).

#### 3.4.3 Changes in the Nrf2 level

**FIGURE 4:**
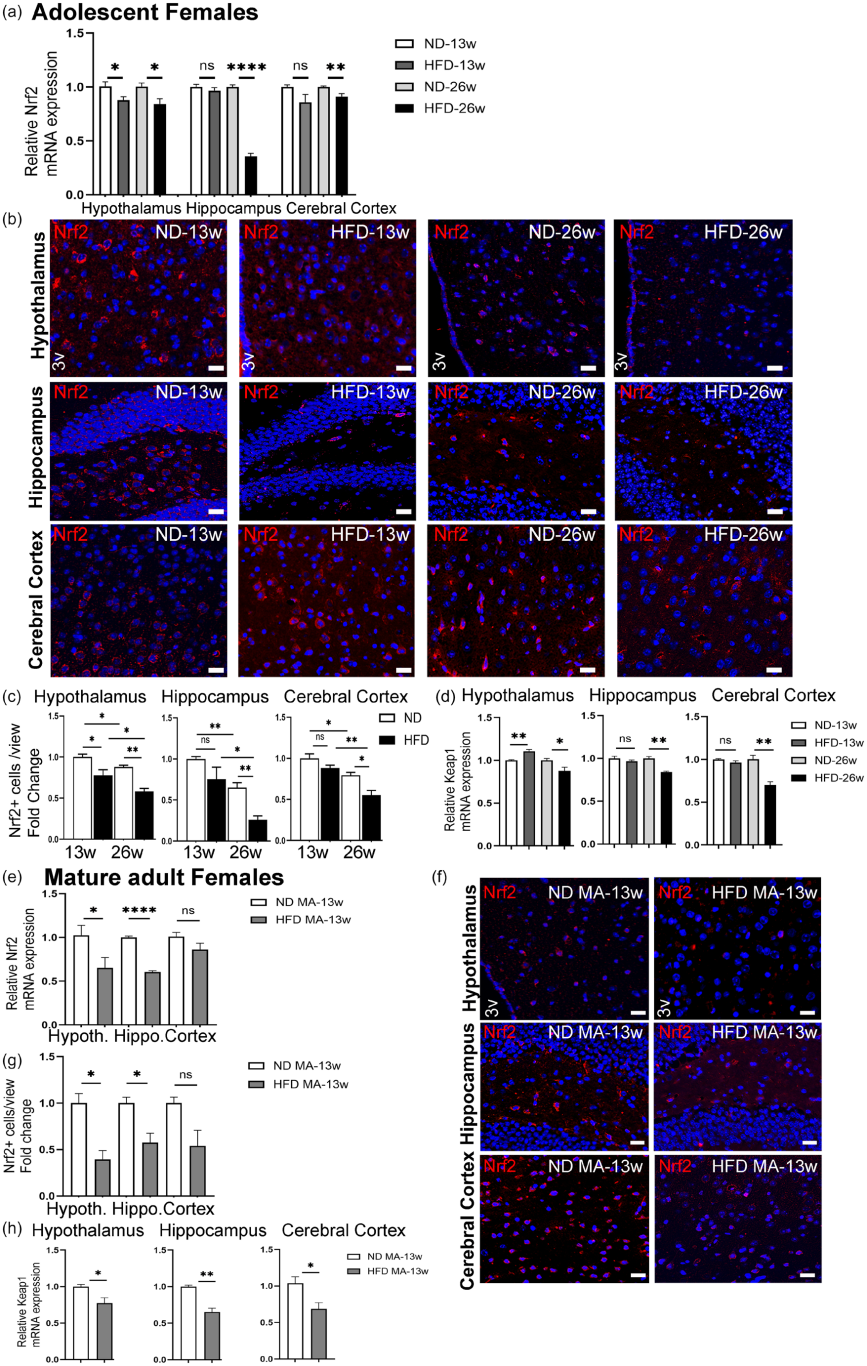
Effect of HFD/obesity on the Nrf2 and Keap1 levels in different brain subregions of female mice. (a) Relative mRNA levels of Nrf2 in the hypothalamus, hippocampus, and cerebral cortex of adolescent female mice fed either ND or HFD for 13 or 26 weeks. (b) IHC analysis of Nrf2‐positive cells in the CNS. Representative images of Nrf2‐positive cells (in red) and Hoechst‐stained nuclei (in blue) were obtained from the hypothalamus region (3v, third ventricle), the hippocampus region, and the cerebral cortex region from mice fed ND or HFD for 13 or 26 weeks. Scale bars: 20 μm. (c) Fold change in Nrf2 positive cell numbers in the hypothalamus, hippocampus, and cerebral cortex regions. (d) Relative mRNA levels of Keap1 in the hypothalamus, hippocampus, and cerebral cortex of adolescent female mice fed either ND or HFD for 13 or 26 weeks. Data are shown as mean ± s.e.m. ns, non‐significant, *p < 0.05, and **p < 0.01. (e) Effect of HFD/obesity on the Nrf2 level in different brain subregions of mature adult (MA) female mice. Relative mRNA levels of Nrf2 in the hypothalamus, hippocampus, and cerebral cortex following 13 weeks of dieting. (f) IHC analysis of Nrf2‐positive cells in different brain sub‐regions. Representative images of Nrf2‐positive cells (in red) and Hoechst‐stained nuclei (in blue) were obtained from the hypothalamus region (3v, third ventricle), the hippocampus region, and the cerebral cortex region from mature adult female mice fed either ND or HFD for 13 weeks. Scale bars: 20 μm. (g) Fold change in Nrf2 positive cell numbers in the hypothalamus, hippocampus, and cerebral cortex regions. (h) Relative mRNA levels of Keap1 in the hypothalamus, hippocampus, and cerebral cortex of mature adult female mice following 13 weeks of dieting. Data are mean ± s.e.m. ns, non‐significant, *p < 0.05, **p < 0.01, and ****p < 0.0001.

Nrf2 is another neurotrophic/neuroprotective factor promoting neuronal antioxidant defense by interacting with Sirt1 and BDNF (Bruna et al., 2018; Huang et al., 2013; Seo & Lee, 2013). Our co‐labeling experiments showed that Nrf2 is expressed in NeuN+ cells (Figure S5C). To determine how Nrf2 expression and the number of Nrf2+ cells in specific brain subregions are affected by HFD, we conducted both RT‐PCR and immunostaining experiments. Our RT‐PCR results showed that after 13 weeks of HFD, Nrf2 expression was significantly reduced in the hypothalamus but not in the hippocampus and cerebral cortex of adolescent females (Figure 4a). In contrast, the prolonged duration (26 weeks) of HFD significantly reduced Nrf2 expression in all three brain subregions. These findings were confirmed by our immunostaining experiments showing that HFD‐fed adolescent females had significantly fewer Nrf2+ cells in their hypothalamus after 13 weeks of diet while displaying no significant changes in their hippocampus or cerebral cortex (Figure 4b,c). Consistent with Nrf2 expression results, the prolonged duration (26 weeks) of HFD significantly reduced the number of Nrf2+ cells in all three brain subregions examined (Figure 4b,c). Notably, a significant reduction in the number of Nrf2+ cells was also observed in all three brain subregions of ND‐fed mice as a result of aging (Figure 4c), suggesting that additional aging of 13 weeks might also have contributed to the observed outcome after the prolonged HFD of 26 weeks.

To further confirm the HFD‐induced changes in the hypothalamus, hippocampus, and cerebral cortex of adolescent females, Nrf2 protein levels were also examined by western blot analysis. After 13 and 26 weeks of HFD, the changes in the Nrf2 protein levels were similar to the mRNA expression and immunostaining results, further substantiating our findings (Figure S6A,C).

When mature adult females were exposed to HFD for 13 weeks, a significant reduction in Nrf2 expression was observed in the hypothalamus and hippocampus compared to their respective controls, while changes in the cerebral cortex remained insignificant (Figure 4e). Similar results were obtained after the immunostaining experiments (Figure 4f,g). However, our western blot analyses revealed significantly reduced Nrf2 protein levels in all three brain regions after 13 weeks of HFD, suggesting a higher impact of HFD on the protein level (Figure S6D,F).

Overall, these findings suggest that age and duration of DIO significantly modulate the level of neurotrophic/neuroprotective factors in different brain subregions.

Nrf2 activity is regulated at different levels including transcriptional, post‐transcriptional, and protein stability. Under homeostatic conditions, cytosolic Kelch‐Like ECH‐Associated Protein 1 (Keap1) regulates Nrf2 activity by binding and ubiquitinating it (Canning et al., 2015; Nguyen et al., 2009; Taguchi et al., 2011; Wakabayashi et al., 2003). Therefore, we also examined how Keap1 expression is affected by HFD /obesity in female brain. As shown in Figure 4d, adolescent mice displayed significantly increased Keap1 expression in the hypothalamus after 13 weeks of HFD while its expression level did not significantly change in the hippocampus and cerebral cortex. The prolonged HFD of 26 weeks resulted in significantly lower Keap1 expression in all three brain subregions of adolescent females with respect to the corresponding controls. Similarly, mature adult females fed HFD for 13 weeks displayed significantly lower Keap1 expression in all three brain subregions (Figure 4h). Since Nrf2‐Keap1 complexing occurs at a 1:2 molar ratio (Horie et al., 2021; Liu et al., 2021; Tong et al., 2006), Keap1 expression may accordingly change along with the Nrf2 expression level. It is possible that upon HFD, Keap1 expression might have been initially increased to accelerate degradation of Nrf2 and then leveled off to maintain the ratio. It is noteworthy to mention that degradation of Nrf2 may also occur independent of Keap1 (Chen et al., 2020; Rada et al., 2011; Rada et al., 2012; Salazar et al., 2006; T. Wu et al., 2014). Further research is needed to address obesity‐associated interactions between Nrf2 and Keap1.

#### 3.4.4 Changes in the superoxide dismutase (SOD) level and activity

**FIGURE 5:**
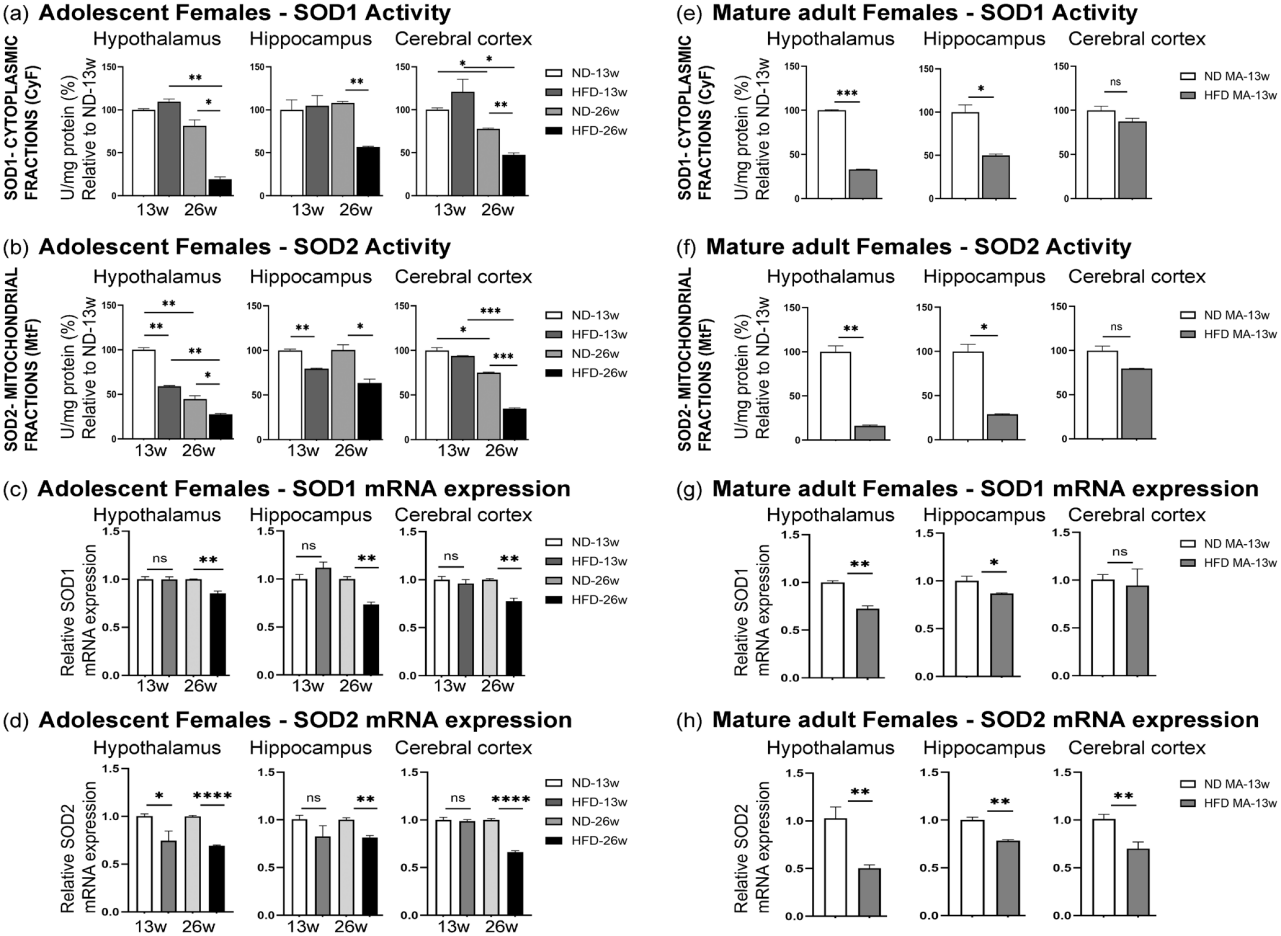
Effect of HFD/obesity on SOD expression and activities in different brain subregions of female mice. Using a colorimetric activity assay, SOD1 and SOD2 activities were analyzed from cytoplasmic and mitochondrial fractions, respectively. The activities of SOD1 (a) and SOD2 (b) were measured in the hypothalamus, hippocampus, and cerebral cortex of adolescent female mice fed either ND or HFD for 13 or 26 weeks. Relative mRNA levels of SOD1 (c) and SOD2 (d) in the hypothalamus, hippocampus, and cerebral cortex of adolescent female mice fed either ND or HFD for 13 or 26 weeks. The activities of SOD1 (e) and SOD2 (f) in the hypothalamus, hippocampus, and cerebral cortex of mature adult (MA) female mice fed either ND or HFD for 13 weeks. SOD activities were normalized by mg protein amount and expressed as relative percentages to their respective ND‐13w. Data are shown as mean ± s.e.m. ns, non‐significant, *p < 0.05, **p < 0.01, and ***p < 0.001. Relative mRNA levels of SOD1 (g) and SOD2 (h) in the hypothalamus, hippocampus, and cerebral cortex of mature adult female mice fed either ND or HFD for 13 weeks. Data are shown as mean ± s.e.m. ns, non‐significant, *p < 0.05, **p < 0.01, and ****p < 0.0001.

The copper/zinc SOD (SOD1) and the manganese SOD (SOD2) are two types of antioxidant enzymes primarily located in the cytoplasm and mitochondria, respectively (Halliwell & Gutteridge, 1995). Nrf2 and Sirt1 are known to induce antioxidant enzymes such as SODs to prevent oxidative stress (Huang et al., 2013). Our immunochemistry staining showing co‐expression of Nrf2 and SOD2 in the same brain cell also suggests the likelihood for such a crosstalk (Figure S5D). To probe how DIO affects the antioxidant system, we isolated both cytoplasmic (SOD1) and mitochondrial (SOD2) fractions from three brain subregions and then assessed SOD activities. Our findings indicate that adolescent female mice exposed to the HFD for 13 weeks displayed comparable SOD1 activity in all three brain subregions with respect to ND‐fed controls (Figure 5a). However, SOD2 activity was significantly lower in the hypothalamus and hippocampus but not in the cerebral cortex (Figure 5b). When adolescent female mice were subjected to a prolonged duration (26 weeks) of HFD, both SOD1 and SOD2 activities were significantly lower in all three brain subregions compared to their respective controls (Figure 5a,b), underlining the marked effect of obesity duration. The SOD1 and SOD2 mRNA expression levels after 13 and 26 weeks of HFD were largely similar to the SOD1 and SOD2 activity patterns except a nonsignificant decrease in SOD2 expression in the hippocampus after 13 weeks of HFD (Figure 5c,d). We also assessed SOD2 protein level by Western blotting and did not observe any significant change after 13 weeks of HFD while the SOD2 protein level was significantly decreased in all three brain subregions after 26 weeks of prolonged HFD (Figure S6G,H).

We next studied SOD activities in mature adult female mice after consumption of an HFD for 13 weeks. As shown in Figure 5e,f, DIO significantly reduced both SOD1 and SOD2 activities in the hypothalamus and hippocampus compared to ND, while the decrease in SOD1 and SOD2 activities remained insignificant in the cerebral cortex. After 13 weeks of HFD, mature adult females displayed SOD1 and SOD2 mRNA expression levels similar to their SOD activity patterns except significantly reduced SOD2 mRNA expression in the cerebral cortex (Figure 5g,h). The SOD2 protein level was also significantly decreased in all three brain subregions (Figure S6I,J).

These findings imply that consuming an HFD for an extended period can compromise the antioxidant system in different regions of the female brain. Additionally, the cerebral cortex seems to be somewhat resistant to HFD‐induced changes.

## 4 DISCUSSION

By subjecting adolescent and mature adult female mice to HFD for different durations and then by investigating the resulting inflammatory and neurotrophic/neuroprotective responses with respect to age‐matched controls fed ND, this study reveals that (1) HFD induces a typical obesity phenotype in female mice independent of their age and duration of HFD; (2) young females display some resistance to obesity‐associated inflammation and initially develop a limited inflammatory response in hypothalamus after an average HFD duration of 13 weeks; (3) extending the duration of HFD in young females to 26 weeks induces a systemic inflammatory response including expression of proinflammatory cytokines in different subregions of CNS and visceral adipose tissue, as well as microgliosis, astrogliosis along with suppression of neurotrophic/neuroprotective factors in all three brain subregions examined; and (4) mature adult females response to an average duration of HFD with systemic inflammation and suppression of neurotrophic/neuroprotective factors in all brain subregions similar to the prolonged HFD, indicating the critical role of age in obesity‐associated comorbidities.

Previous studies clearly demonstrated that obesity is associated with chronic inflammation leading to different comorbidities, including diabetes, cardiovascular diseases, osteoarthritis, and neurodegenerative diseases (Bastien et al., 2014; Boles et al., 2017; Mazon et al., 2017; Park et al., 2014; Pi‐Sunyer, 2009; Rachoń & Teede, 2010). While most of the DIO studies were conducted in males, a small number of studies performed in both genders suggested a sexual dimorphism in response to HFD‐induced weight gain, fat distribution, regulation of energy balance, neurogenesis, and brain fatty acid composition (Bruder‐Nascimento et al., 2017; Hwang et al., 2010; Ingvorsen et al., 2017; Pettersson et al., 2012; Robison et al., 2020; Rodriguez‐Navas et al., 2016). Compared to males, female mice seem to have reduced susceptibility to obesity‐induced inflammation and to respond slower and lesser extent to HFD (Hong et al., 2009; Pettersson et al., 2012; Sanchez et al., 2024; Stranahan et al., 2023; Yang et al., 2014). This sexual dimorphism has been attributed to the difference in the level of circulating gonadal steroid hormones that also change in females depending on the phase of a reproductive cycle (Bardhi et al., 2023; Camporez et al., 2013; Hong et al., 2009; Jones et al., 2000). In the present study, we attempted to minimize steroid hormone‐dependent variations by inducing synchronized ovulation in all females and collecting tissue samples 14 h later. This approach also allowed us to collect ovulated oocytes and thus to separately study effects of DIO on fertility. To make our fertility study more relevant to infertility of women who are overweight and delay their childbearing years up to mid‐thirties, we started HFD of mature adult females at the age of 14 weeks and ended it at 27 weeks. Overall, our findings on the response of adolescent females to an average duration (13 weeks) of HFD are consistent with the results of the aforementioned studies in terms of reduced susceptibility to obesity‐induced inflammation, while our experiments involving a longer duration (26 weeks) of HFD in adolescent females and average duration of HFD in adult females further reveal that both age and duration of DIO modify the inflammatory responses in females. Since we used adolescent and mature adult female mice in our experiments, the observed DIO‐induced inflammatory response in females is unlikely due to reproductive senescence. Hence, the protective effects of gonadal steroids in females might be progressively declining by persisting obesity and increasingly compromised homeostasis due to ongoing low‐level inflammatory processes and aging as well. This concept is also supported by the findings of an earlier study (Salinero et al., 2018).

Considering the global epidemic status of obesity and associated comorbidities, our findings regarding the decisive effects of age and duration of obesity are of significance. In particular, our results showing that adolescent females are resistant to proinflammatory processes during an average duration of obesity are encouraging and suggest that adverse effects of obesity might be avoided/minimized by early corrective interventions. The importance of the early interventions was also underlined by the next set of experiments showing that even adolescent females respond to the persisting obesity with marked systemic inflammatory processes, including increased expression of proinflammatory cytokines, microgliosis, astrogliosis, and suppression of neurotrophic/neuroprotective factors in all brain regions examined. In this regard, it is worth noting that the prolonged duration of HFD is ultimately associated with the progression of age, of which decoupling is hardly possible. Hence, aging might also have contributed to the observed effects of prolonged HFD. Moreover, our experimental data indicate that as age progresses, female mice become more susceptible to obesity‐associated proinflammatory processes even after an average duration (13 weeks) of DIO, further underlining the importance of early corrective interventions.

Regardless of age and duration of HFD consumption, we observed a significant weight gain and hypertrophied adipocytes in all female mice. These results are generally in agreement with published data (Kesherwani et al., 2015; Krishna et al., 2016; Winzell & Ahrén, 2004). Our findings of significantly increased expression of TNFα, IL1β, and IL 22 in visceral adipose tissue after a prolonged period of HFD are also in line with a previous study reporting elevated levels of IL1β and TNFα in the peri‐ovarian fat pad after 7 months of HFD (Nteeba et al., 2013). The increased IL 22 level, as observed in the present study, seems to play a critical role in obesity‐mediated systemic inflammation (Dalmas et al., 2014; Kim et al., 2019) that may, in turn, amplify neuroinflammation and cognitive decline (Erion et al., 2014; Lee & Mattson, 2014).

Astrogliosis and microgliosis represent another aspect of neuroinflammation. Published data indicate that upon HFD intake, microglia and astrocytes accumulate within the hypothalamus and play a crucial role in HFD‐induced neuroinflammation by producing cytokines and inducing inflammatory responses (Baufeld et al., 2016; De Souza et al., 2005; Sugiyama et al., 2020; Valdearcos et al., 2014). It has been suggested that microglia in the hypothalamus are the initial responders to the HFD by producing mediators that ultimately activate astrocytes (Douglass et al., 2017; Valdearcos et al., 2014), a process referred to as reactive astrogliosis and characterized by hypertrophy and hyperplasia (Berkseth et al., 2014; Buckman et al., 2013; Thaler et al., 2012). Our Iba1 and GFAP expression data and related immunohistochemistry results obtained after subjecting adolescent females to 13 weeks of HFD also suggest the hypothalamus as an early target, although the hippocampus also displayed increased expression of Iba1 and GFAP (Table S2). However, unlike in the hypothalamus, a significant increase in the expression of proinflammatory cytokines in the hippocampus was observed only after a prolonged duration of (26 weeks) of HFD or advanced age. Hence, our findings support previous short‐term DIO studies suggesting that the hypothalamus is likely to be an early target for inflammation (Dalvi et al., 2017; Thaler et al., 2012, 2013).

**FIGURE 6:**
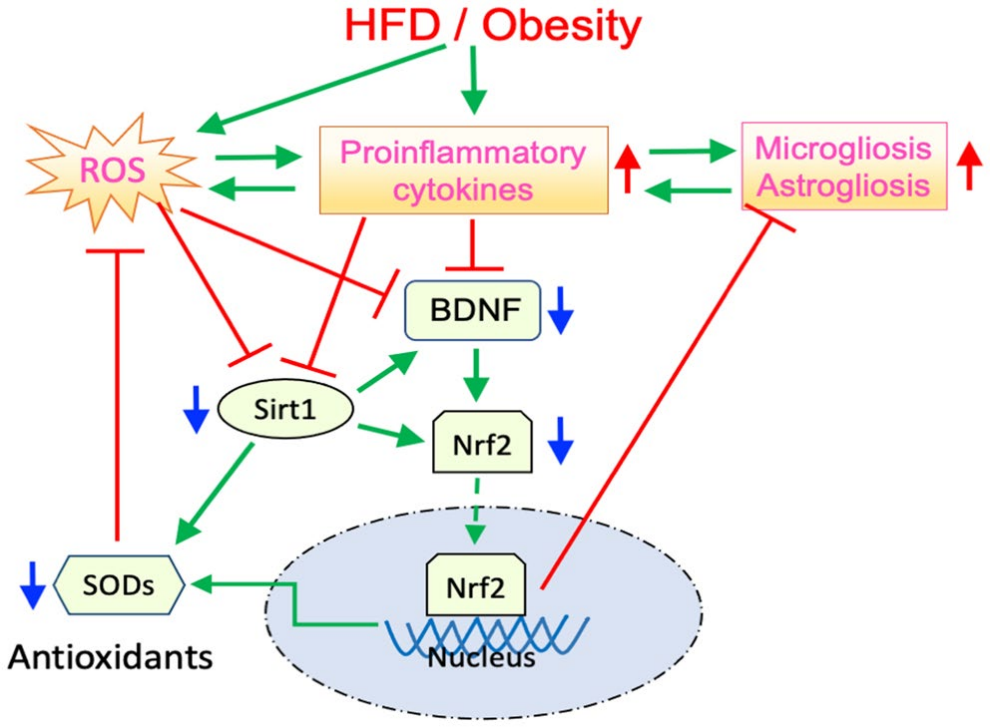
A simplified model of HFD/obesity‐induced neuroinflammation showing possible interactions along with the main outcomes of the present study. Consumption of HFD and the resulting obesity lead to increased production of both reactive oxygen species (ROS) and proinflammatory cytokines (green arrows), initially in the hypothalamus and subsequently in other brain subregions in an age‐ and time‐dependent manner. The increase in the production of ROS and proinflammatory cytokines initiates a positive feedback loop between them (green arrows in both directions). Overproduction of proinflammatory cytokines also triggers microgliosis and astrogliosis in the hypothalamus, resulting in another positive feedback loop (green arrows in both directions). These events promote proinflammatory processes in other brain subregions, ultimately culminating in neuroinflammation. Both Sirt1 and BDNF activate Nrf2 (green arrows) that translocate into the nucleus and bind to the ARE sequence of different antioxidant/cytoprotectant genes to induce their transcription. Consequently, both excessive ROS formation and microgliosis/astrogliosis are suppressed (red blunt arrows). However, ROS and proinflammatory cytokines induced by sustained HFD/obesity inhibit both Sirt1 and BDNF (red blunt arrows) and thus impair the Nrf2‐dependent upregulation of antioxidant/cytoprotectant genes, resulting in chronic neuroinflammation. The related results of the study are depicted as upward red and downward blue arrows to indicate upregulation and downregulation, respectively. BDNF, brain‐derived neurotrophic factor; HFD, high‐fat diet; Nrf2, nuclear factor erythroid 2‐related factor 2; Sirt1, Sirtuin1; SODs, superoxide dismutases.

The molecular mechanism of obesity‐associated comorbidities is complex and has not been fully understood despite considerable progress in the past two decades (Gregor & Hotamisligil, 2011; Hotamisligil, 2006; Lee & Mattson, 2014; Rohm et al., 2022). A growing body of experimental evidence suggests causative roles of oxidative stresses and neuroinflammation in the underlying mechanism of obesity‐induced brain injury (Boitard et al., 2014; De Souza et al., 2005; Miller & Spencer, 2014). A partial and simplified model, including our relevant findings, is presented in Figure 6 for neuroinflammation. The excess food intake and/or HFD seem to activate a cascade of inflammatory pathways involving toll‐like receptors (TLRs), c‐jun N‐terminal kinase (JNK), an inhibitor of κ kinase (IKK), protein kinase R (PKR), and nuclear factor‐kappa B (NF‐κB), which increase expression of proinflammatory cytokines such as TNF‐α, IL1β, and IL6 (De Souza et al., 2005; Hirosumi et al., 2002; Milanski et al., 2009; Posey et al., 2009; Uysal et al., 1997). Another HFD‐induced early event entails an increase in mitochondrial β‐oxidation of excessive free fatty acids (not shown) (Chen, Du, et al., 2021; Ly et al., 2017; Rosca et al., 2012; Tan & Norhaizan, 2019), tipping the balance in ROS formation/elimination. The resulting elevated ROS production increases the expression of proinflammatory cytokines and thus initiates a positive feedback loop, ultimately ending in neuroinflammation. In addition, the initial increase in proinflammatory cytokine expression in the hypothalamus is likely to trigger microgliosis and astrogliosis, as observed in the present study, leading to the establishment of another positive feedback loop. As a result of a sustained HFD/obesity, the initial inflammatory processes in the hypothalamus may induce microgliosis and astrogliosis in other brain subregions. In fact, signs of microgliosis in the hippocampus and cerebral cortex seem to start earlier than a significantly increased expression of proinflammatory cytokines in the same subregions (Table S2), supporting the role of the hypothalamic microgliosis in amplification of inflammatory processes in the hippocampus and cerebral cortex. Furthermore, our findings, in line with published data, also suggest that HFD induces alterations in adipose tissue, resulting in increased expression of proinflammatory cytokines (Gao et al., 2015; Hotamisligil et al., 1993; Nteeba et al., 2013; Shu et al., 2012). Hence, significantly elevated ROS and proinflammatory cytokines may suppress the expression of neurotrophic/neuroprotective factors such as Sirt1 and BDNF (Figure 6). As a matter of fact, our results, along with published data (Heyward et al., 2012; Molteni et al., 2002; Wu et al., 2004) reinforce such an outcome, although no significant reduction of BDNF has also been reported in one study (Heyward et al., 2012). As an epigenetic modifier, Sirt1 may induce a multitude of effects, including neurotrophic and neuroprotective ones, by upregulating BDNF, Nrf2, and SODs (Figure 6) (Cao et al., 2018; Caruso et al., 2021; Ding et al., 2016; Huang et al., 2013; Jeong et al., 2011; Nimmagadda et al., 2013; Zocchi & Sassone‐Corsi, 2012). Likewise, BDNF plays a marked role in sustaining brain function through inducing neurogenesis, synaptic plasticity, anti‐inflammatory, and prosurvival actions, while its downregulation may lead to neuronal death (Lima Giacobbo et al., 2019; Waterhouse & Xu, 2009). Both Sirt1 and BDNF activate Nrf2 (Bruna et al., 2018), which, in turn, upregulates antioxidant defense in the brain (Chan & Kwong, 2000; Itoh et al., 1997; Kensler et al., 2007; Seo & Lee, 2013). Under normal circumstances, Nrf2 is constitutively ubiquitinated and degraded by binding to cytosolic Kelch‐Like ECH‐Associated Protein 1 (Keap1) (Nguyen et al., 2009; Taguchi et al., 2011; Wakabayashi et al., 2003). Once activated, unbound Nrf2 molecules accumulate in the nucleus (Bruna et al., 2018; Kensler et al., 2007), where they bind to antioxidant response elements (AREs) in the promoters of different genes (Itoh et al., 1997; Kensler et al., 2007; Motohashi & Yamamoto, 2004), resulting in transcription of several antioxidants such as SODs. Hence, inadequate activation of Nrf2 by suppressed Sirt1 and BDNF levels, as observed in the present study, may lead to insufficient expression of antioxidants and thus, to low‐level chronic neuroinflammation when HFD/obesity persists. In line with previous studies (Cao et al., 2018; Sun et al., 2018), our results indeed show significantly reduced activity of cytoplasmic SOD1. Moreover, our study reveals that the activity of mitochondrial SOD2 is even more suppressed by HFD/obesity. Considering that mitochondria represent sites for substantial ROS generation, downregulation of mitochondrial SOD2 is likely to significantly contribute to proinflammatory processes. Nrf2 also seems to downregulate microgliosis and astrogliosis independent of redox homeostasis by directly inhibiting proinflammatory cytokine expression (Kobayashi et al., 2016; Rojo et al., 2010). Thus, reduced activation of Nrf2 may not be sufficient to effectively stop microgliosis and astrogliosis, resulting in sustained inflammatory processes. Overall, our results align with the presented partial model and support the notion that unless intervened, HFD/obesity‐associated neuroinflammation may persist because of compromised antioxidant defense and inefficient suppression of proinflammatory cytokines due to the downregulated Sirt‐1‐BDNF‐Nrf2 axis. Further research is needed to verify causal interactions and to elucidate the involvement of complementary pathways.

The current study has also some limitations in terms of addressing (1) if the partially and fully developed obesity‐associated proinflammatory processes could be reversed by correcting diet (e.g., switching to ND after subjecting adolescent and mature adult females to HFD for 13 weeks) or by inhibiting prominent proinflammatory cytokines and (2) if the composition of HFD, such as the ratio of saturated fats to non‐saturated ones, replacing long‐chain triglycerides with medium‐chain triglycerides, or altering carbohydrate and amino acid content/composition may influence the outcome. In respect to the former, encouraging results have been reported by two different studies involving female mice (Sims‐Robinson et al., 2016; Sobesky et al., 2014). Sobesky et al. were able to attenuate HFD‐induced cognitive impairment and neuroinflammation by dietary switch from HFD to ND and blocking IL‐1β with an IL‐1 receptor antagonist while Sims‐Robinson et al. ameliorated HFD‐associated memory deficit and impaired hippocampal insulin signaling by dietary reversal. Concerning the latter, two studies in male rats and mice have reported that fat composition (lower saturated fat content and changing its source to coconut oil or a low omega6/omega3 ratio) appears to modulate HFD‐induced neuroinflammation and impaired behavior (Maric et al., 2014; Sanchez et al., 2024). Further studies are needed to address to what extent obesity‐associated comorbidities are reversible and to develop effective interventions.

In conclusion, young female mice are partially resistant to HFD/obesity‐induced inflammation for an average exposure period, while the prolonged duration of HFD/obesity and/or advanced age make female mice prone to obesity‐associated neuroinflammation. From the translational perspective, the findings of the present study highlight the importance of early interventions to combat obesity and, thus, to avoid the associated comorbidities.

## AUTHOR CONTRIBUTIONS

B.E. and A.E. designed the study. B.E. conducted the experiments and analyzed the data. All authors contributed to the writing and editing of the manuscript.

## CONFLICT OF INTEREST STATEMENT

None declared.

## Supporting information


Appendix S1.

## Data Availability

Data available on request from the authors.
