# Supplementary material for: Age and duration of obesity modulate the inflammatory response and expression of neuroprotective factors in mammalian female brain
Source: Aging Cell. 2024 Sep 4;23(12):e14313. doi: 10.1111/acel.14313 (PMC11634740; doi:10.1111/acel.14313)

**Table S1:** Primer pairs used for gene expression analysis by RT-PCR.

| **Gene** | **Forward Primer (5’-3’)** | **Reverse Primer (5’-3’)** |
| --- | --- | --- |
| **TNFα** | CCTGTAGCCCACGTCGTAG | GGGAGTAGACAAGGTACAACCC |
| **IL1β** | GAAATGCCACCTTTTGACAGTG | TGGATGCTCTCATCAGGACAG |
| **IL6** | CTGCAAGAGACTTCCATCCAG | AGTGGTATAGACAGGTCTGTTGG |
| **IL22** | ATGAGTTTTTCCCTTATGGGGAC | GCTGGAAGTTGGACACCTCAA |
| **GFAP** | CGGAGACGCATCACCTCTG | TGGAGGAGTCATTCGAGACAA |
| **Iba1** | GGATTTGCAGGGAGGAAAAG | TGGGATCATCGAGGAATTG |
| **Bdnf** | TCATACTTCGGTTGCATGAAGG | AGACCTCTCGAACCTGCCC |
| **Nrf2** | TCTTGGAGTAAGTCGAGAAGTGT | GTTGAAACTGAGCGAAAAAGGC |
| **Sirt1** | AGCAACATCTCATGATTGGCACCG | TCTGCCACAGCGTCATATCATCCA |
| **Keap1** | TGCCCCTGTGGTCAAAGTG | GGTTCGGTTACCGTCCTGC |
| **SOD1** | AACCAGTTGTGTTGTCAGGAC | CCACCATGTTTCTTAGAGTGAGG |
| **SOD2** | CAGACCTGCCTTACGACTATGG | CTCGGTGGCGTTGAGATTGTT |
| **Ppia** | CGCGTCTCCTTCGAGCTGTTTG | TGTAAAGTCACCACCCTGGCACAT |

**Table S2**: Overview of the experimental outcomes. Adolescent (5 weeks old) and mature adult (14 weeks old) female mice were subjected to HFD for either 13 or 26 weeks, whereas aged-matched females receiving ND served as controls. Visceral adipose tissue was analyzed for inflammatory cytokines, while different brain regions were evaluated for inflammatory cytokines, microgliosis, astrogliosis, and neuroprotection. Horizontal green arrows indicate no significant changes, while upward red and downward blue arrows show significant increases and decreases, respectively.

**Adolescence – 13 weeks of HFD**

| Abdominal -Adipose tissue | **→** TNFα | **→** IL1β | **→** IL6 | **→** IL22 |  |
| --- | --- | --- | --- | --- | --- |
| Brain-Inflammation | | | | | |
| Hypothalamus | **↑** TNFα | **↑** IL1β | **→** IL6 | **↑** Iba1 | **↑** GFAP |
| Hippocampus | **→** TNFα | **→** IL1β | **→** IL6 | **↑** Iba1 | **↑** GFAP |
| Cerebral cortex | **→** TNFα | **→** IL1β | **→** IL6 | **↑** Iba1 | **→** GFAP |
| Brain-Neuroprotection | | | | | |
| Hypothalamus | **↓** Sirt1 | **→** BDNF | **↓** Nrf2 | **→** SOD1 | **↓** SOD2 |
| Hippocampus | **↓** Sirt1 | **→** BDNF | **→** Nrf2 | **→** SOD1 | **→** SOD2 |
| Cerebral cortex | **→** Sirt1 | **→** BDNF | **→** Nrf2 | **→** SOD1 | **→** SOD2 |

**Adolescence – 26 weeks of HFD**

| Abdominal -Adipose tissue | **↑** TNFα | **↑** IL1β | **→** IL6 | **↑** IL22 |  |
| --- | --- | --- | --- | --- | --- |
| Brain-Inflammation | | | | | |
| Hypothalamus | **↑** TNFα | **↑** IL1β | **→**IL6 | **↑** Iba1 | **↑** GFAP |
| Hippocampus | **↑** TNFα | **↑** IL1β | **→**IL6 | **↑** Iba1 | **↑** GFAP |
| Cerebral cortex | **↑** TNFα | **↑** IL1β | **↑** IL6 | **↑**  Iba1 | **↑** GFAP |
| Brain-Neuroprotection | | | | | |
| Hypothalamus | **↓** Sirt1 | **↓** BDNF | **↓** Nrf2 | **↓** SOD1 | **↓** SOD2 |
| Hippocampus | **↓** Sirt1 | **↓** BDNF | **↓** Nrf2 | **↓** SOD1 | **↓** SOD2 |
| Cerebral cortex | **↓** Sirt1 | **→** BDNF | **↓** Nrf2 | **→** SOD1 | **↓** SOD2 |

**Mature Adult – 13 weeks of HFD**

| Abdominal -Adipose tissue | **↑** TNFα | **↑** IL1β | **↑** IL6 | **↑** IL22 |  |
| --- | --- | --- | --- | --- | --- |
| Brain-Inflammation | | | | | |
| Hypothalamus | **↑** TNFα | **↑** IL1β | **→** IL6 | **↑** Iba1 | **↑** GFAP |
| Hippocampus | **↑** TNFα | **↑** IL1β | **↑** IL6 | **↑** Iba1 | **↑** GFAP |
| Cerebral cortex | **↑** TNFα | **↑** IL1β | **→** IL6 | **↑** Iba1 | **↑** GFAP |
| Brain-Neuroprotection | | | | | |
| Hypothalamus | **↓** Sirt1 | **↓** BDNF | **↓** Nrf2 | **↓** SOD1 | **↓** SOD2 |
| Hippocampus | **↓** Sirt1 | **↓** BDNF | **↓** Nrf2 | **↓** SOD1 | **↓** SOD2 |
| Cerebral cortex | **↓** Sirt1 | **↓** BDNF | **→** Nrf2 | **→** SOD1 | **→** SOD2 |

**Figure S1:** Development of obesity in adolescent female C57BL/6 mice subjected to HFD for 13 or 26 weeks. **A)** Weekly average body weights (g) on ND (open circles) or HFD (closed black circles) are shown for 13 and 26 weeks (black arrows), with a significant difference of *p*<0.0001 between the two groups. The red arrow indicates when the difference in the mean body weight was significant. **B)** Representative pictures of mice fed ND or HFD for 13 or 26 weeks. **C)** The daily food intake is shown as grams per mouse (n=6). **D)** The daily energy intake is shown as kcal per mouse and calculated by multiplying food intake with 5.49% kcal/g for HFD and 3.1 kcal/g for ND (n=6). **E)** Representative pictures of H&E-stained subcutaneous WAT displaying adipocyte morphology after 13 weeks (13w) and 26 weeks (26w) on ND and HFD. The insets show macrophage infiltrations known as crown-like structures (CLS). Scale bars: 50 μm. **F)** Quantification of adipocyte size of subcutaneous WAT obtained from mice fed ND or HFD for 13 or 26 weeks. Adipocyte area was measured using Adiposoft software. **G)** Quantification of CLS per 100 adipocytes in the subcutaneous WAT obtained from adolescent mice fed ND or HFD for 13 or 26 weeks. Data are shown as mean ± s.e.m. (n>5). ns, non-significant, * *p*<0.05, *** *p*<0.001, and **** *p*<0.0001.


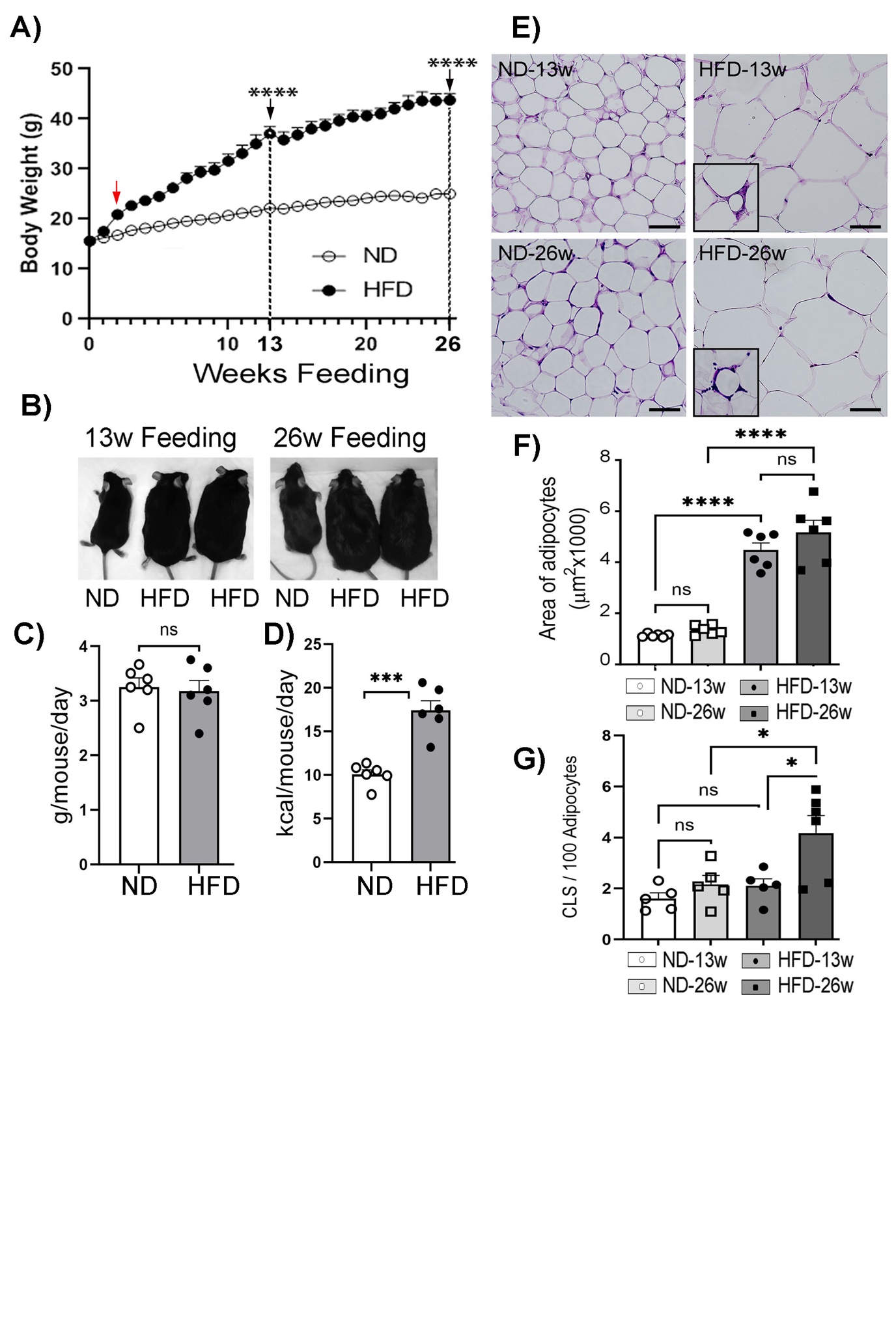


**Figure S2:** Development of obesity in mature adult (MA) female C57BL/6 mice subjected to HFD for 13 weeks. **A)** Weekly average body weights (g) on ND (open squares) or HFD (closed black squares) are shown for 13 weeks, with a significant difference of *p*<0.0001 between the two groups (black arrow). At the end of dieting, both groups displayed a significantly increased average body weight. **B)** Representative pictures of mature adult female mice fed ND or HFD for 13 weeks. **C)** The daily food intake is shown as grams per mouse (n=5). **D)** The daily energy intake is shown as kcal per mouse (n=5). **E)** Representative pictures of H&E-stained subcutaneous WAT displaying adipocyte morphology after ND and HFD of 13 weeks. The insets show macrophage infiltrations known as CLS. Scale bars: 50 μm. **F)** Quantification of adipocyte size of subcutaneous WAT obtained from mature adult female mice fed ND or HFD for 13 weeks. **G)** Quantification of CLS per 100 adipocytes in the subcutaneous WAT obtained from mature adult mice fed ND or HFD for 13 weeks. Data are shown as mean ± s.e.m. (n>5). * *p*<0.05, *** *p*<0.001, and **** *p*<0.0001.


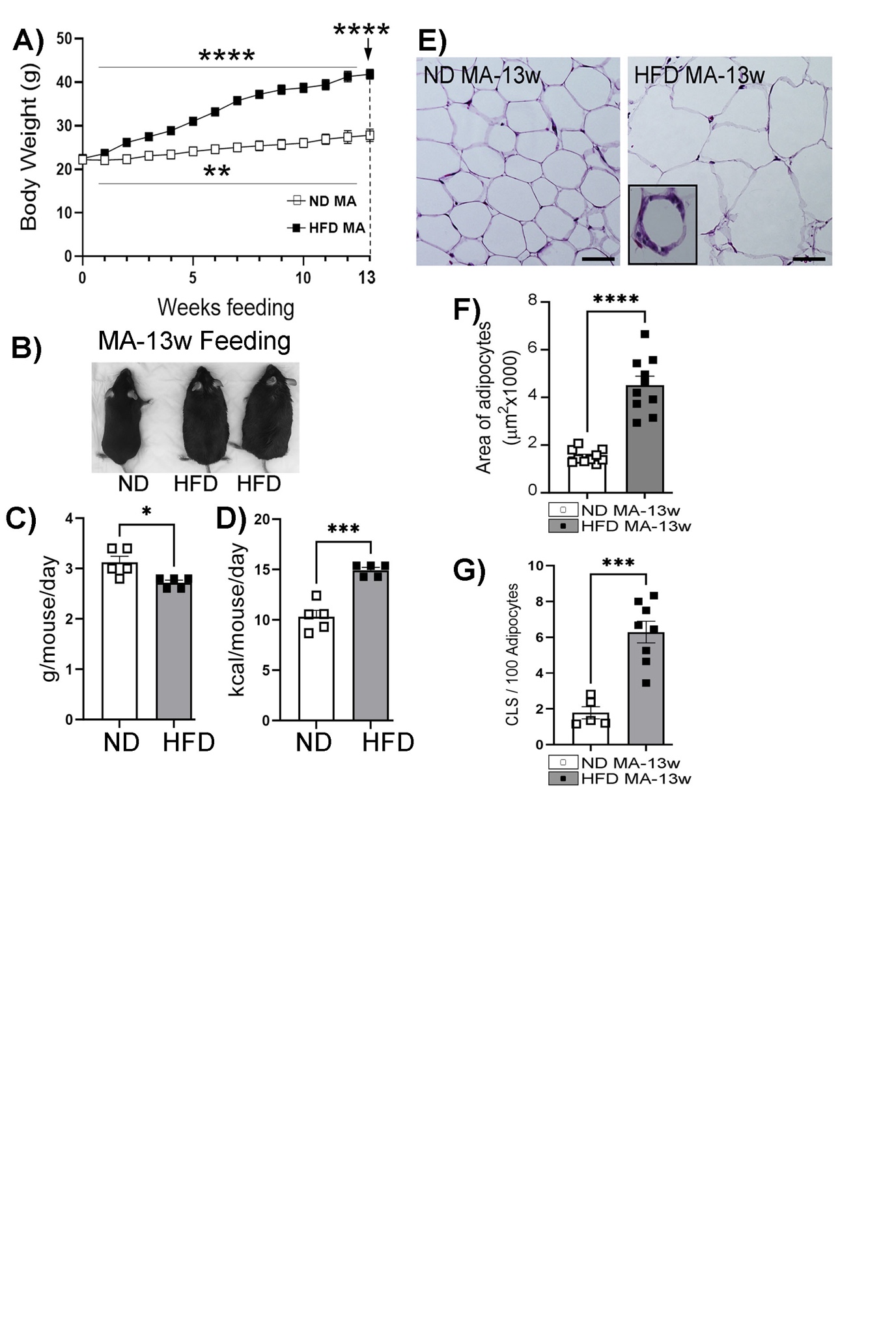


**Figure S3:** HFD/obesity-induced microgliosis in different brain subregions of female mice. **A)** IHC analysis of microglia cells in the CNS. Representative images of Iba1+ (microglia marker) cells (in red) and Hoechst-stained nuclei (in blue) in the hypothalamus region (3v, third ventricle), the hippocampus region, and the cerebral cortex region of adolescent female mice fed either ND or HFD for 13 or 26 weeks. Scale bars: 20 μm. **B)** Fold changes in Iba1+ cell numbers in the hypothalamus, hippocampus, and cerebral cortex of adolescent female mice fed either ND or HFD for 13 or 26 weeks. **C)** Fold changes in activated microglia in the hippocampus region of adolescent female mice fed either ND or HFD for 26 weeks. Representative images of Iba1+ resting and activated microglia (in red) and Hoechst-stained nuclei (in blue). Scale bar: 5 μm. Data are shown as mean ± s.e.m. ns, non-significant, * *p*<0.05, and ** *p*<0.01. **D)** Representative images of Iba1+ cells (in red) and Hoechst-stained nuclei (in blue) in the hypothalamus region (3v, third ventricle), the hippocampus region, and the cerebral cortex region of mature adult (MA) mice fed either ND or HFD for 13 weeks. Scale bars: 20 μm. **E)** Fold changes in Iba1+ cell numbers in the hypothalamus, hippocampus, and cerebral cortex of adult female mice fed either ND or HFD for 13 weeks. Data are shown as mean ± s.e.m. * *p*<0.05 and ** *p*<0.01.

**
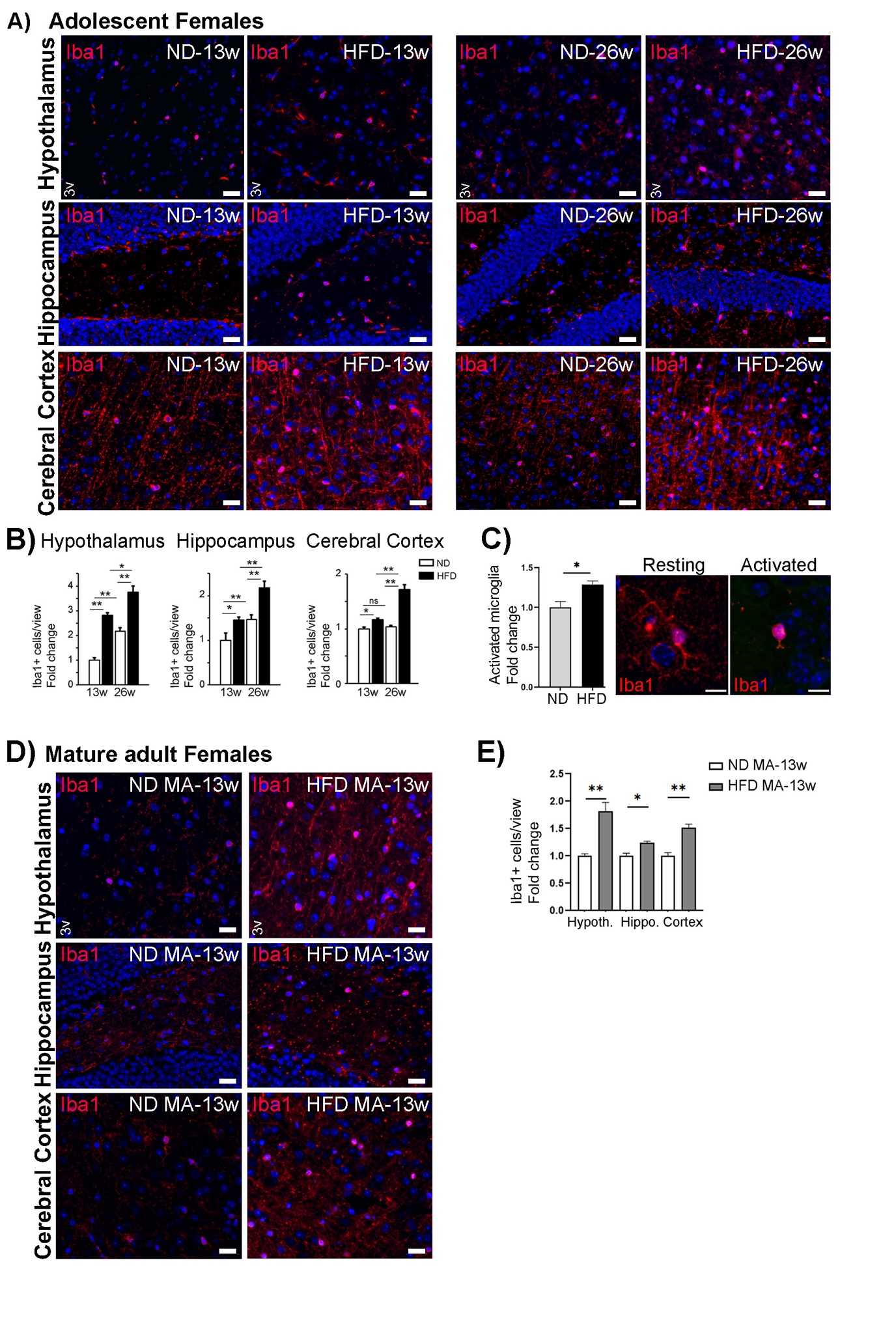
**

**Figure S4:** HFD/obesity-induced astrogliosis in different brain subregions of female mice. **A)** IHC analysis of astroglia cells in the CNS. Representative images of GFAP+ (astrocyte marker) cells (in red) and Hoechst-stained nuclei (in blue) in the hypothalamus region (3v, third ventricle), the hippocampus region, and the cerebral cortex region of adolescent female mice fed either ND or HFD for 13 or 26 weeks. Scale bars: 20 μm. **B)** Fold changes in GFAP+ cell numbers in the hypothalamus, hippocampus, and cerebral cortex of adolescent female mice fed either ND or HFD for 13 or 26 weeks. Data are shown as mean ± s.e.m. ns, non-significant, * *p*<0.05, ** *p*<0.01, *** *p*<0.001, and **** *p*<0.0001. **C)** Representative images of GFAP+ cells (in red) and Hoechst-stained nuclei (in blue) in the hypothalamus region (3v, third ventricle), the hippocampus region, and the cerebral cortex region from mature adult (MA) mice fed either ND or HFD for 13 weeks. Scale bars: 20 µm. **D)** Fold changes in GFAP+ cell numbers in the hypothalamus, hippocampus, and cerebral cortex of adult female mice fed either ND or HFD for 13 weeks. Data are shown as mean ± s.e.m. * *p*<0.05.


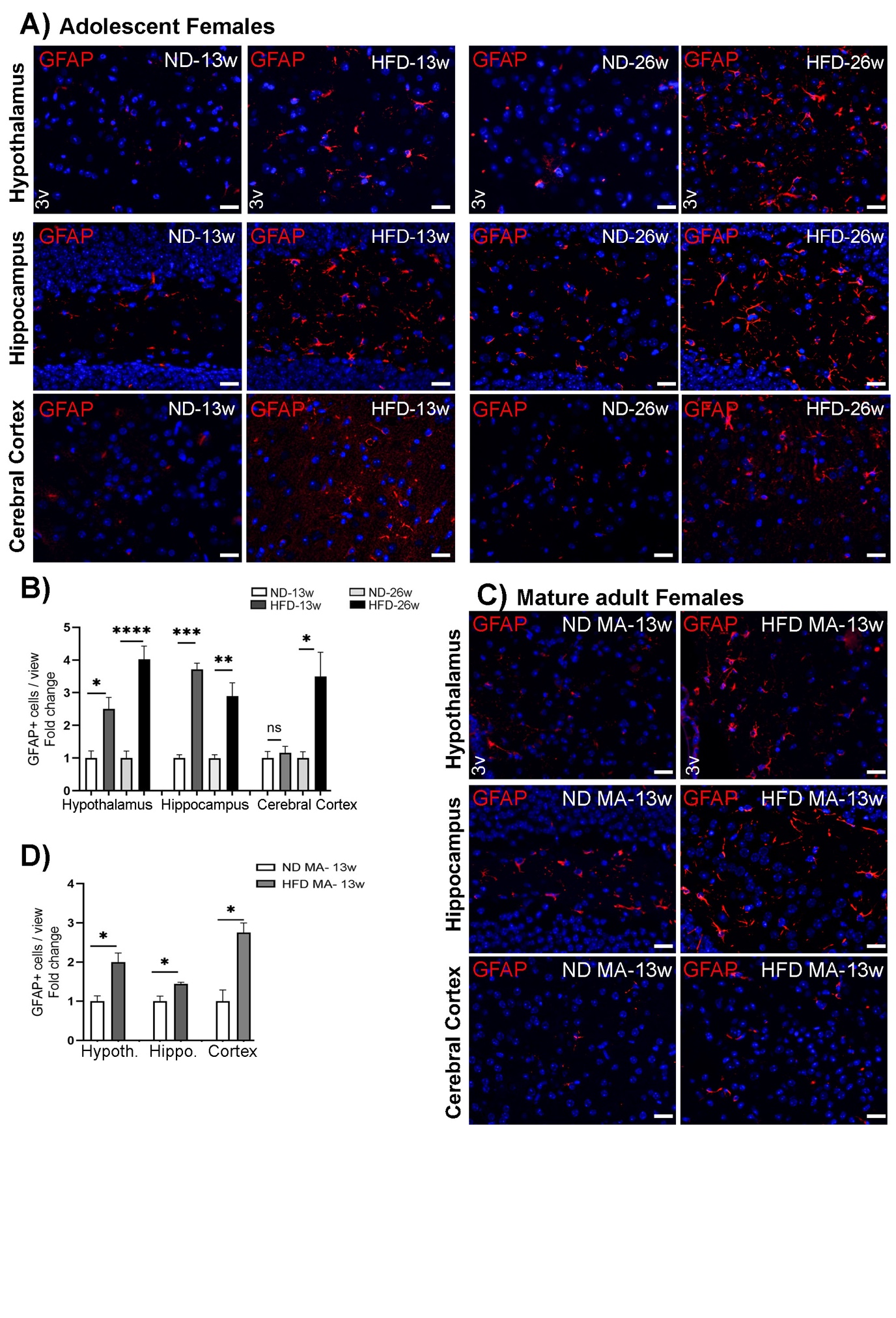


**Figure S5:** Representative images showing co-expression of neuronal marker NeuN (in green) with **A)** BDNF (in red), **B)** Sirt1 (in red), and **C)** Nrf2 (in red) in the hippocampus of adolescent females fed ND for 26 weeks. Cell nuclei are stained with Hoechst (in blue). **D)** Representative images showing co-expression of SOD2 (in green) and p-Nrf2 S40 (in red) in the same cell located in the hippocampus of adolescent females fed ND for 26 weeks. Double immunostaining was performed using SOD2 and Nrf2 phospho-S40 antibodies while cell nucleus was stained with Hoechst (in blue). Scale Bars: 10µm.


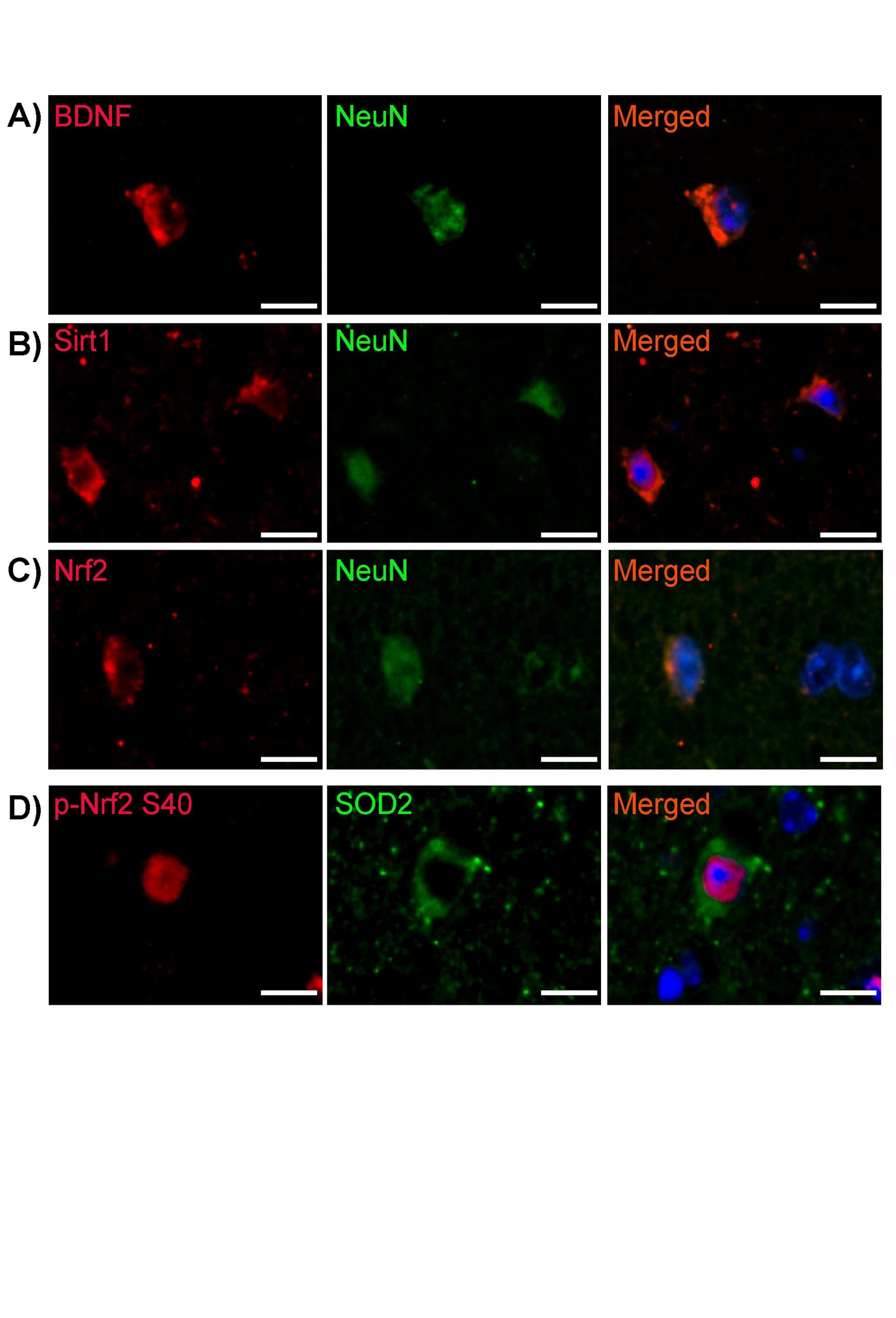


**Figure S6:** Effect of HFD/obesity on protein levels of Nrf2, Sirt1, and SOD2 in different brain subregions of female mice. **A)** Representative western blot images show the protein expression levels of Nrf2 and Sirt1 in different brain subregions of adolescent female mice fed either ND or HFD for 13 or 26 weeks. β-actin was used as a loading control. **B)** Bar graphs depicting quantitative Sirt1 protein levels in adolescent female mice. **C)** Bar graphs showing quantitative Nrf2 protein levels in adolescent female mice. **D)** Representative western blot images show the protein expression levels of Nrf2 and Sirt1 in different brain subregions of mature adult female mice fed either ND or HFD for 13 weeks. β-actin was used as a loading control. **E)** Bar graphs depicting quantitative Sirt1 protein levels in mature adult female mice. **F)** Bar graphs showing quantitative Nrf2 protein levels in mature adult female mice. Data are shown as mean ± s.e.m. ns, non-significant, * *p*<0.05, and ** *p*<0.01. **G)** Representative western blot images show the protein expression level of SOD2 in different brain subregions of adolescent female mice fed either ND or HFD for 13 or 26 weeks. β-actin was used as a loading control. **H)** Bar graphs depicting quantitative SOD2 protein levels in adolescent female mice. **I)** Representative western blot images show the protein expression level of SOD2 in different brain subregions of mature adult female mice fed either ND or HFD for 13 weeks. β-actin was used as a loading control. **J)** Bar graphs depicting quantitative SOD2 protein levels in mature adult female mice.


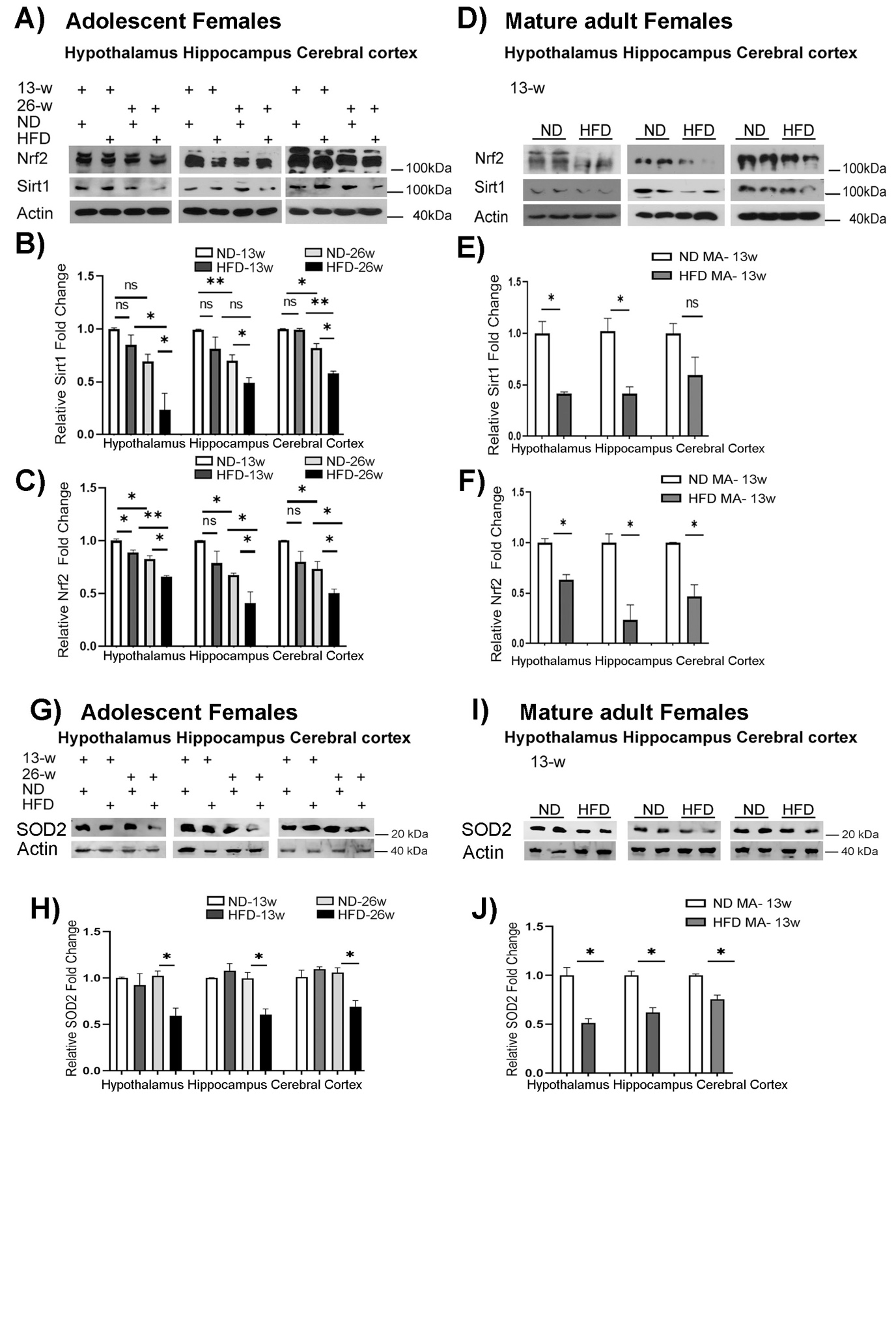

Supplement: Supplementary file 1 — Appendix S1. [file ACEL-23-e14313-s001.docx]
